# *DISPAQ*: Distributed Profitable-Area Query from Big Taxi Trip Data †

**DOI:** 10.3390/s17102201

**Published:** 2017-09-25

**Authors:** Fadhilah Kurnia Putri, Giltae Song, Joonho Kwon, Praveen Rao

**Affiliations:** 1 Department of Big Data, Pusan National University, Busan 46241, Korea; fadhilahkp@pusan.ac.kr; 2 School of Computer Science and Engineering, Pusan National University; Busan 46241, Korea; gsong@pusan.ac.kr; 3 Department of Computer Science & Electrical Engineering, University of Missouri-Kansas City, Kansas City, MO 64110, USA; raopr@umkc.edu

**Keywords:** taxi trip data, GPS sensors, profitable areas, distributed processing, PQ-index, Z-skyline, big data

## Abstract

One of the crucial problems for taxi drivers is to efficiently locate passengers in order to increase profits. The rapid advancement and ubiquitous penetration of Internet of Things (IoT) technology into transportation industries enables us to provide taxi drivers with locations that have more potential passengers (more profitable areas) by analyzing and querying taxi trip data. In this paper, we propose a query processing system, called Distributed Profitable-Area Query (*DISPAQ*) which efficiently identifies profitable areas by exploiting the Apache Software Foundation’s Spark framework and a MongoDB database. *DISPAQ* first maintains a profitable-area query index (PQ-index) by extracting area summaries and route summaries from raw taxi trip data. It then identifies candidate profitable areas by searching the PQ-index during query processing. Then, it exploits a Z-Skyline algorithm, which is an extension of skyline processing with a Z-order space filling curve, to quickly refine the candidate profitable areas. To improve the performance of distributed query processing, we also propose local Z-Skyline optimization, which reduces the number of dominant tests by distributing killer profitable areas to each cluster node. Through extensive evaluation with real datasets, we demonstrate that our *DISPAQ* system provides a scalable and efficient solution for processing profitable-area queries from huge amounts of big taxi trip data.

## 1. Introduction

Internet of Things (IoT) technology enables interconnections between large volumes of distributed and heterogeneous smart devices allowing them to communicate seamlessly with users. Recently, IoT devices such such as sensors, global positioning systems (GPSs), and cameras have become widely used in transportation industries. For example, several countries such as the USA [1], Germany [2], Japan [3] and Korea [4], collect diverse data from taxis equipped with IoT devices. Data science includes the effective translation of data into novel insights, discoveries and solutions [5]. Big data analytics as a big part of data science enables us not only to provide intelligent services to customers, but also to improve work efficiency and profitability of taxi drivers by analyzing the collected data.

Finding good taxi strategies for improving services and profits is one of the core applications in smart transportation [6]. Most existing approaches analyze collected GPS sensor data to extract taxi strategies, e.g., increasing traffic system efficiency [7], measuring graph-based efficiency of taxi services [8], understanding service strategies such as searching for passengers, passenger delivery, and service area preference [6], plus finding good locations based on minimum cruising time [9,10,11], maximum profit [12], minimum cruising distance [10] and/or high passenger demand [13,14,15,16,17]. Broadly speaking, we believe that these approaches are intended to find high-profit locations (occasionally, we use the terms “high-profit locations” and “profitable areas” interchangeably) for taxi drivers although different methods have been proposed.

For passenger search-strategy improvements, a great deal of research has been done on finding profitable areas [9,13,14,15,16,17,18,19,20]. However, we observed that most of the existing solutions, which are based on clustering techniques [13,14,16,17] or statical techniques such as autoregressive integrated moving average (ARIMA) [10], chi-square distribution [18], statistical learning [11], predictive distribution [15] and probability model [19], only consider one or two factors for finding profitable areas, although it is well known that various factors influence finding profitable areas. A profitability map approach [9] and a recommendation system approach [20] utilize multiple factors to find profitable areas. However, all existing approaches utilize a relatively small amount of taxi trip data, which fits into the memory of one machine.

A motivating example could intuitively illustrate the challenges for finding profitable areas.

**Figure 1:**
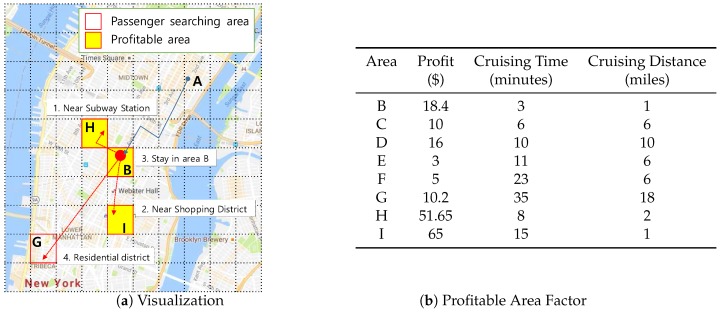
Finding profitable areas.

**Example** **1.** Consider a New York City taxi driver working in the areas shown in Figure 1. The taxi driver picks up a passenger in area A and delivers him to area B. After dropping the passenger in area B, he wants to find a new passenger, by either staying in area B or going to another area. For simplicity, we assume that the driver has four candidate profitable areas in which to search for new passengers: (1) move to area H near a subway station, (2) move to area I near a shopping district, (3) stay in area B, or (4) move to area G near a residential district.

In this example, we assume that three factors affect finding profitable areas: (1) profit, (2) cruising time, and (3) cruising distance. Figure 1b describes the example values for each candidate profitable area. If we consider profit factor only, then area I is the best location, since it has the highest profit. Area B could qualify as the best location when we consider both cruising time and cruising distance. If we consider three factors simultaneously, areas B, H, and I should be considered profitable areas. We see that all values for area G are worse than those of areas B, H, and I. Thus area G cannot be a profitable area. However, we cannot decide which one is better among areas B, H, and I. This is a typical scenario for the skyline query processing approach [21].

In addition, we can provide better suggestions to taxi drivers if a profitable area query system relies on their past experiences as recorded in taxi trip data. Then, it is necessary to compute huge volumes of taxi trip data, because the amount of data increases quickly, especially with the numerous taxis that are active in a big metropolitan city.

In order to build an efficient and scalable profitable areas query system, we need to address the following three challenges motivated by Example 1: (1) efficiently answer profitable-area queries, (2) find profitable areas by considering several factors simultaneously, and (3) deal with huge volumes of taxi trip data.

To address the above challenges, we propose a query processing system, called *DISPAQ*, which facilitates parallel processing by combining the Apache Software Foundation’s Spark framework [22,23] and a MongoDB database [24]. First, to answer profitable-area queries efficiently, we devise a spatial-temporal data structure, which is called a profitable-area query index (PQ-index). The PQ-index is a hash-based index that consists of two major components: (1) spatio-temporal hash keys and (2) extended route summaries. An extended route summary is combinations of area summaries and route summaries, where an area summary contains beneficial information about the area and a route summary manages expense information from the route. A query processor in *DISPAQ* utilizes the PQ-index to obtain candidate profitable areas. Second, we consider the problem of finding profitable areas with multiple factors under skyline query processing [21]. However, a pairwise point-to-point dominance test in skyline processing is a time-consuming process, so we exploit a Z-skyline method [25] which uses a Z-order space filling curve to cluster data points into blocks. The Z-skyline approach can guarantee refining candidate profitable areas by checking for dominance. A dominated area means that all values of the factors of the area are worse than others. Third, to deal with large volumes of taxi trip data, we propose distributed processing to retrieve final profitable areas. Thus, the construction of the PQ-index and the Z-skyline approach are implemented as a distributed way by using Spark and MongoDB. In addition, we devised an optimized shuffling of block-pruning data, which maximizes dominated-area elimination by sending killer areas to every node in the cluster.

This paper is an extended version of our previous publication [26]. We extend our previous work as follows: First, we provide a complete formal definitions for a profitable-area query. Second, we present comprehensive algorithms for constructing the PQ-index and distributed processing of profitable-are queries. In addition, we discuss the proofs of theorems to validate the correctness of the algorithms and the complexity of the distributed algorithms. Finally, we perform experiments to demonstrate the efficiency of the *DISPAQ* system. We conduct an extensive performance evaluation with real taxi trip data sets from New York City and Seattle, USA.

Overall, the main contributions of this paper as a crucial part of data science can be summarized as follows:

- We proposed a distributed profitable-area query process system, called *DISPAQ*, for huge volumes of taxi trip data. The main goal of *DISPAQ* is to provide valuable profitable area information to users, which is one of main activities of data science.
- To quickly retrieve candidate profitable areas, *DISPAQ* organizes multiple factors about a profitable area into a spatial-temporal index called PQ-index. We define and extract multiple factors from the raw taxi trip dataset collected GPS sensors.
- *DISPAQ* executes an efficient Z-skyline algorithm to refine candidate profitable areas. The Z-skyline algorithm could reduce unnecessary dominance tests and avoid pairwise dominant tests. The Z-skyline approach is implemented as a distributed algorithm to manage big taxi trip data.
- We propose an optimized method for distributed Z-Skyline query processing by sending killer areas to each node, which maximizes the filtering of dominated areas.
- We conduct extensive experiments on a large scale two real datasets from New York City and Chicago to determine the efficiency and effectiveness of *DISPAQ*. We compared our Z-Skyline query processing method with two basic skyline methods (block-nested looping and divide-and-conquer) in a distributed approach. The experimental results show that our approach outperforms the existing methods.

The remainder of this paper is organized as follows. Section 2 describes the related research work, and Section 3 provides the preliminaries of *DISPAQ*. Section 4 provides the design of PQ-index and the detailed steps for constructing it. In Section 5, we explain how to process a profitable-area query by exploiting the Z-skyline algorithm using a combination of Spark and MongoDB. Section 6 presents the performance evaluation results and a comparison of *DISPAQ* with its competitors. Finally, Section 7 concludes the paper.

## 2. Related Work

In this section, we briefly survey existing approaches and highlight their differences compared to our *DISPAQ* system. We broadly group the approaches into three categories based on functionality: (1) taxi passenger searching strategy, (2) taxi information data structure, and (3) skyline query.

### 2.1. Taxi Passenger Searching Strategies

As one of the crucial goals of taxi drivers is to carry as many passengers as possible, a variety of ways of finding highly profitable areas or recommending hot spots of taxi users have been suggested. Roughly, we can categorize the previous work into four categories: (1) clustering-based approaches, (2) statistical approaches, (3) specialized model approaches and (4) machine learning-based approaches. An extensive survey on mining taxi GPS traces can be found in [27].

In the first category, previous solutions extracted patterns of taxi drivers and predicted high-profit areas or routes as the result of passenger searching [13,14,16,28]. Lee et al. [13] utilized a K-means clustering method to extract hot-spots from historical GPS taxi data. To discover the taxi demand distribution and predict hot-spots, the iTaxi system studied the effects of three clustering methods: K-means clustering, agglomerative hierarchal clustering, and density-based spatial clustering of applications with noise (DBSCAN) [14]. Zhang et al. [28] proposed a novel spatio-temporal clustering algorithm to recommend top-5 high-profit pickup areas. Recently, an improved DBSCAN algorithm [16] was proposed to recommend hot spot-based routes by analyzing short-dated GPS sensor data. However, the above research considered only the passenger demand when recommending high-profit areas, and did not consider the big data issues when dealing with large volumes of GPS sensor data.

In the second category, several research projects built prediction models for the passenger search problem [10,11,15,29,30,31,32]. An improved auto-regressive integrated moving average (ARIMA) scheme [10] forecasts high taxi-passenger-demand spots by using GPS traces. To predict the spatio-temporal distribution of taxi passenger demand, an online recommendation system based on time series forecasting techniques is proposed [29]. The same authors proposed a short-term time series prediction model for the number of services at a given taxi stand using streaming data [30]. T-Finder [11] is another recommendation system for both taxi drivers and passengers, which exploits a probabilistic model constructed from GPS trajectories of taxis. An incremental ARIMA model [15] predicts high passenger-demand spots by employing a learning model based on historical GPS data. Dong et al. [31] proposed a recommendation system by using linear equations to compute the score of the each road segment. To find out the max-score cruising route, they also uses a skyline computation to reduce the search space. However, they focused on recommending routes not profitable area and did not touch the issues of big data. SCRAM [32] aims to provide recommendation fairness for a group of competing taxi drivers. It utilized the expected driving cost (EDC) function with complex event probabilities. The above-mentioned methods mainly regard taxi trip data as time series and suggest recommendation systems based on time series prediction models. However, *DISPAQ* focuses on the profitable-area query processing which requires to efficiently manage a huge volumes of big taxi trip data. The distinctive feature of *DISPAQ* system is that it returns a set of profitable areas not just one profitable area.

In the third category, several specialized models [9,20] are utilized for determining the next cruising location. Powell et al. [9] defined a profitability score to construct a spatial-temporal profitability map. Their system suggests profitable locations to reduce the cruising time of taxicabs by using a fixed complex profitability formula. However, they do not tackle the issues when dealing with huge volumes of taxi trips. Our *DISPAQ* system also uses the concept of the profitability map. However, *DISPAQ* first constructs a PQ-index from raw taxi trip data. The distributed construction algorithm is proposed to handle a huge volumes of taxi trips. The profitability map of *DISPAQ* includes candidate profitable areas by searching the PQ-index. By exploiting the PQ-index, *DISPAQ* can efficiently reduce the search spaces. Then, the distributed skyline query processing method is applied to the profitability map to refine candidate profitable areas. Due to the skyline concept, *DISPAQ* returns a set of comparable profitable areas not just one profitable area. Recently, two location-to-location graph models [20] such as an OFF-ON model and an ON-OFF model were adopted to recommend the next cruising location by considering three factors. Although these two model considers three factors, they mainly relies on the transition probability from one location to another location. When dealing with huge volumes of taxi trips, two graph models cannot fit into a memory, thus the performance will be degraded. However, this method do not tackle this big data issue when dealing with huge volumes of taxi trips.

In the fourth category, several machine learning based approaches have been studied [33,34,35,36]. Time series analysis techniques based on non-homogeneous Poisson processes are utilized to predict short-term approximate local probability density functions of taxi stands [33]. DeepSD [35] exploits a novel deep neural network structure for short-term prediction on the gap between the car-hailing supply and demand in a certain area. A reinforcement learning based system [36] is developed to learn from real trajectory logs of taxi drivers and to recommend the profitable locations to the drivers. PRACE [34] is a deep learning based taxi recommender system for finding passengers. It executes a prediction task as a multi-classification problem rather than a regression problem. As mentioned in the paper [37], deep learning technologies are good at predicting over uncertain events. Since our research is conducted based on a profitable-area query processing system, thus we mainly focus on the efficient distributed algorithms which utilize the PQ-index and the skyline query concept. However, we believe that the above-mentioned deep learning methods could supplement our *DISPAQ*.

The goal of our *DISPAQ* system is similar to the aforementioned studies. However, our approach is different from the existing work in the following aspects: (1) We build a PQ-index for maintaining profitable area-related information. (2) We extend skyline query processing to retrieve profitable areas by considering multiple factors. (3) We devise a distributed algorithm for handling huge volumes of taxi trip data.

### 2.2. Taxi Information Data Structure

Another related topic of this paper is to build efficient data structures for handling and analyzing taxi information. Generally speaking, tree-based index, hash-based index or specialized data structures are exploited to efficiently maintain taxi information.

Several research attempts have been made to manage taxi information based on tree-based or hash-based indexes [38,39,40,41,42,43,44]. An adaptive quadtree [40] was used to store a trajectory data set, and a combination of BPR-Quadtree and a minhash index [41] was built for storing historical trajectory data. A kd-tree was utilized to provide passengers with expected fare and trip duration [39] or to visualize New York City taxi trips by treating each taxi trip as a point in a k-dimensional space [38]. A light-weight spatial index based on geohash [42] was constructed to answer basic spatial queries such as *containing*, *containedIn*, *intersects* and *withinDistance*. The authors implemented the geohash index on San Francisco taxi traces. T-Share [43] is a taxi ride-sharing service that uses a spatio-temporal grid index to store an ordered taxi list in a location based on distance and arrival time. Huang et al. [44] suggested a kinetic tree to dynamically match realtime trip requests to servers in a road network to allow real-time ridesharing. A GPU-based index [45] was proposed to support complex spatio-temporal queries over large, histrorical data, which is a generalization of the kd-tree. The complex spatio-temporal queries are basically select-from-where style queries which efficiently utilize the GPU-based index. However, the core operation of profitable-area queries is the dominance test which requires not the generalized GPU-based index but the specialized PQ-index proposed in this paper.

At the second category, several specialized data structures are devised to efficiently manage taxi information [46,47,48,49,50]. Nanocube [46] is a in-memory data cube structure for easily generating visual encodes such as heatmaps, histograms, and parallel coordinate plots from spatio-temporal datasets including taxi trips. However, it was only designed to answer queries from interactive visualization systems, thus it does not allow profitable-are queries. A frequent trajectory graph [47] was invented to handle trajectory information for finding areas of high taxi-passenger demands. The querying and extracting timeline information system [48] builds a timeline query index (TQ-index) to manage traffic information according to a timeline model. A time-evolving origin-destination (O-D) matrix [49] deals with a continuous stream of GPS traces and maintains accurate statistics of interests. The O-D matrix focuses on monitoring the evolution of urban dynamics from GPS traces, whereas *DISPAQ* was designed to provide a distributed profitable-area query system. *SigTrac* [50] extracts traffic matrices from traffic sensor data and exploits a singular value decomposition (SVD) technique to process only traffic similarity queries.

Our *DISPAQ* system constructs a specialized index structure called a PQ-index. The PQ-index consists of extended route summaries, which are combinations of area and route summaries from raw taxi trip data to efficiently answer profitable-area queries. In addition, different from the above approaches, *DISPAQ* could build and utilize the PQ-index in a distributed way for handling huge amounts of taxi trip data from GPS sensors.

### 2.3. Distributed Skyline Query Processing

Since *DISPAQ* extends a skyline query processing algorithm to support profitable area queries, we briefly explain research efforts in distributed skyline query processing.

Several researchers have proposed processing skyline queries in a distributed way [51,52,53]. Afrati et al. [52] investigated parallel skyline processing based on a massively parallel (MP) model that requires the data to be perfectly load-balanced. A novel, enhanced distributed dynamic skyline (EDDS) technique [51] was proposed and implemented for wireless sensor networks. Zhou et al. [53] investigated probabilistic skyline queries over uncertain data in distributed environments. These researchers proposed solutions based on their own models, whereas *DISPAQ* utilizes the distributed processing functionalities of Spark [22,23] to answer profitable-area queries.

Some researchers focused on computing skyline queries using MapReduce framework [54,55,56,57,58]. Generally, MapReduce-based skyline processing consists of two parts: (1) computing local skylines and (2) finding global skylines. Since centrally finding global skylines from local skylines would bottleneck the whole process, various partitioning techniques were proposed. Zhang et al. implemented MapReduce-based block-nested looping (MR-BNL), MapReduce-based sort-filter skyline (MR-SFS), and MapReduce-based bitmap (MR-Bitmap) approaches [54]. MR-BNL and MR-SFS showed better performance in most cases, although they don’t work well for high dimensional data due to point-to-point dominance tests. An MR-Angle approach [55] used grid partitioning of the data space to reduce the processing time when selecting optimal skyline objects. A SKY-MR method [57] built a sky-quadtree and a risky-quadtree to effectively prune non-skylines and non-reverse skylines. This pruning method also has a role in load-balancing computations. Mullesgaard et al. designed a grid-partitioning technique to divide data space into partitions, and represented each partition as a bitstring [56]. The bitstring helps prune partitions that cannot have skyline tuples. Recently, Koh et al. [58] proposed dominator reduction rules for limiting the number of dominance tests, and a data sample-selection algorithm for optimizing a local skyline process.

Our *DISPAQ* system is different from the aforementioned approaches in the following aspects. First, we focus on retrieving profitable areas based on distributed skyline query processing. Second, we obtain candidate profitable areas by exploiting the PQ-index which limits the search space. Third, we utilize a Z-Skyline algorithm to refine candidate profitable areas. Due to a monotonic ordering property and an automatic clustering property, Z-skyline avoids unnecessary dominant tests and pairwise dominance tests among profitable areas.

## 3. Preliminaries

In this section, we first present the frequently used notations in the paper. Then, we provide basic definition of taxi trip data and explain the overall architecture of *DISPAQ* system.

### 3.1. Notations

For reference, Table 1 shows our frequently used notation. Each definition will reintroduced when first used in the paper.

### 3.2. Taxi Trip Data

Recently in many urban cities taxis have been equipped with GPS sensors for recording trip information. Thus, we conducted our study on two real-world datasets collected in New York City and the City of Chicago. Table 2 shows a snippet of the New York City taxi trip dataset. Each row in the dataset describes a distinct taxi trip including locations, time stamps and taxi fare information.

The formal definition of a taxi trip is as follows.

**Definition** **1.** *(Taxi trip) Each taxi trip T is denoted as an 8-tuple* $\left(t_{p},t_{d},l_{p},l_{d},d,fa,tia,toa\right)$*, where* $t_{p}$ *and* $l_{p}$ *refer to the pickup time/location at the beginning of a trip,* $t_{d}$ *and* $l_{d}$ *are the drop-off time/location at the end of the trip, d means the distance of the trip,* fa *is the fare amount,* tia *is the tip amount, and* toa *is the tolls amount.*

Since large numbers of taxi trips contain wide variations of GPS coordinates, we utilize geohash [59] to divide geographic regions into a hierarchical structure. Geohash separates areas based on grid cells using Z-order curve, which enables us to divide and merge areas (regions) elastically. Thus, we can easily compute aggregate values in the region due to the characteristics of the geohash and reduce the computation time for obtaining aggregated values in the region.

We formally define an area based on geohash as follows.

**Definition** **2.** *(Area) An area is regarded as a group of exact locations, which is defined as* $ar=geohash(l_{lat},l_{long},len)$*, where* $l_{lat}$ *and* $l_{long}$ *are latitude and longitude of a location, and* len *is the length of geohash code for the area.*

Note that a location is an exact point for a taxi trip and an area means a region that might include several taxi trips.

As we can see, each taxi trip has two locations: pickup and drop-off. Generally, the actual route of a taxi trip requires many GPS coordinates from the pickup area to the drop-off area. However, in this paper, we define a route, rt, of each taxi trip as the pair (pickup area, drop-off area). Since an area is denoted as a geohash code, route rt is also represented by two geohash codes.

**Definition** **3.** *(Route) A route* (rt) *is represented as a 2-tuple* $\left(ar_{o},ar_{d}\right)$*, where* $ar_{o}$ *is an origin area and* $ar_{d}$ *is a destination area.*

The aforementioned definitions are explained in Example 2.

**Example** **2.** *The first row (the first taxi trip* $T_{1}$*) in Table 2 has (−73.98278, 40.75492) as the pickup location and (−73.18142,40.68773) as the drop-off location. Assume that the length of the geocode is 7. This means each area covers approximate 150 m × 150 m region. If we apply the geohash algorithm, then the geohash values of the pickup location and drop-off location are represented as* dr5ryp *and* dr5ryn*, respectively. Thus, the route of* $T_{1}$ *is a pair of origin and destination areas (*dr5ryp*,* dr5ryn*). To simplify area and route for another example, we change area* $ar_{B}$ *as B and route* $rt_{dr5ryp-dr5ryn}$ *as* B−G*. This notation and visualization are illustrated in Figure 1.*

### 3.3. Architecture Overview

**Figure 2:**
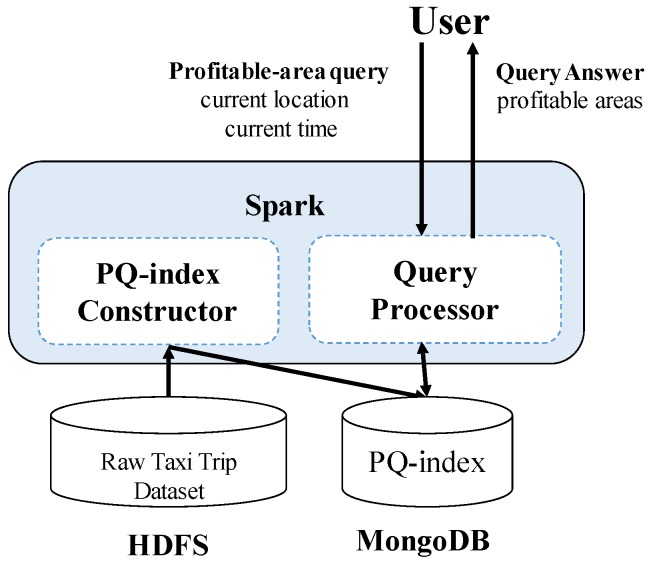
High-level architecture of Distributed Profitable-Area Query (*DISPAQ*).

Figure 2 shows the high-level architecture of *DISPAQ*. The key components of *DISPAQ* are the PQ-index constructor, the query processor, the Hadoop Distributed File System (HDFS) and the MongoDB document store. The PQ-index constructor transforms raw taxi trip into the aggregated values, then builds a PQ-index based on area information and route information. The query processor executes a profitable-area query with the current location and time from a user. The results are returned to the user in two steps: (1) a profitability map computation phase and (2) a refinement phase for pruning candidate profitable areas. *DISPAQ* exploits the parallel processing of Spark and a MongoDB NoSQL document store: HDFS stores the raw taxi trip data and the MongoDB stores and utilizes the PQ-index.

**Figure 3:**
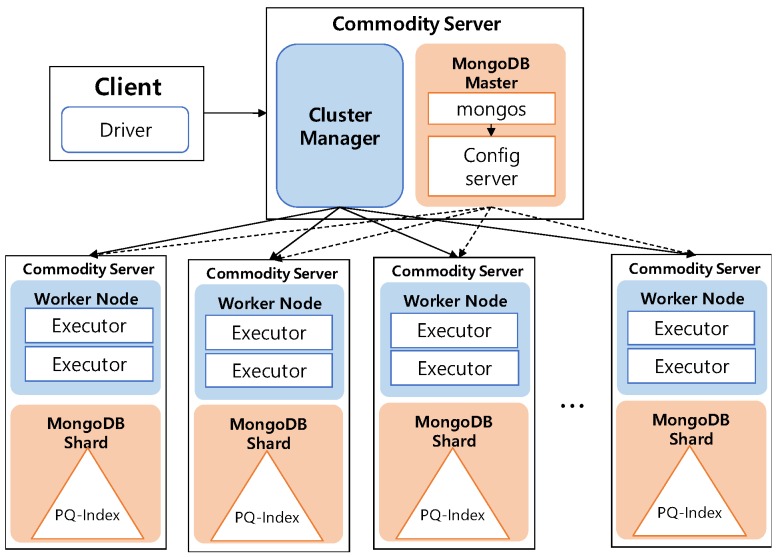
Distributed components of *DISPAQ*.

One of the key characteristics of *DISPAQ* is distributed processing of profitable-area queries by combining Spark and the MongoDB document store. Figure 3 depicts three main physical components of *DISPAQ*: (1) the client, (2) a commodity server as Spark Master and (3) commodity servers as work nodes. The profitable-area query processing mainly relies on Spark. The client has a Spark driver application, which receives a profitable query from a user and sets a Spark configuration. The Spark driver manages the job flow, schedules tasks, and is available the entire time the application is running. When the configuration is completed, the configuration information is sent to one of the commodity servers that includes a cluster manager in Spark working as a master node. The other commodity servers working as slave nodes have executors, which are responsible for executing work in the form of tasks, as well as for storing any data. Specifically, these executors construct a PQ-index and also execute profitable-area queries. MongoDB stores the PQ-index across commodity servers (shards). Thus, one of the commodity servers becomes a MongoDB master (mongos) and a config sever at the same time. The other servers are MongoDB shards, which store a subset of the PQ-index.

With this high-level overview of the system, we now explain the process of PQ-index construction and processing of profitable area queries.

## 4. Constructing a Profitable Area Query Index

This section presents our profitable area query index (PQ-index) and explains how to build a PQ-index from raw taxi trip data.

### 4.1. Components of the PQ-Index

Our *DISPAQ* system executes a profitable-area query in two steps: (1) collecting candidate profitable areas into a profitability map and (2) refining the candidate areas via extended skyline query processing. Since the values of raw taxi trip data can change dynamically, depending on the current time and location, it is difficult to obtain a profitability map immediately without checking all possible candidates. The intuition in the PQ-index is to pre-compute all possible combinations of candidate areas before executing a user query.

**Figure 4:**
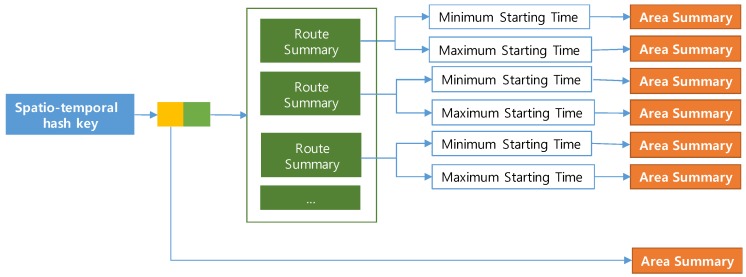
A conceptual view of a PQ-index.

A PQ-index is a hash-based spatio-temporal index structure that maintains aggregated taxi trip information for retrieving candidate profitable areas efficiently. The PQ-index consists of three major components: (1) a spatio-temporal hash key, which helps to quickly identify aggregated taxi trip information; (2) an area summary, which contains calculated profits from an (origin) area at a particular time; and (3) extended route summaries, which are combinations of route summaries and (destination) area summaries for managing computed profits of routes in an area. The profits are calculated by considering average benefits and expenses of routes from the area. Figure 4 depicts a logical (conceptual) design of the PQ-index. As explained, a spatio-temporal hash key has two main elements connected by two pointers: (1) an orange box connecting to an origin area summary and (2) a green box connecting to extended route summaries. We now describe each of these four PQ-index components in detail.

#### 4.1.1. Spatio-Temporal Hash-Key Definition

The PQ-index has a spatio-temporal hash key as a pair (time period, area code): an area code records the geohash code of a location; and a fixed time interval is used as the time period. Since a profitable area needs two input parameters, as explained in Definition 7, we decide the pair (time period, area code) as a hash-key of the PQ-index. For each spatio-temporal hash-key, the PQ-index stores computed profits of routes and an area into an extended route summary.

An area code and a time period are used as major input parameters for the summarization because the aggregated values differ from one area to another at different times. As explained in Definition 2, we use a geohash code to denote a specific group of locations, since areas are static. On the other hand, a time period is a dynamic feature, which should be determined after analyzing raw taxi trip data.

**Figure 5:**
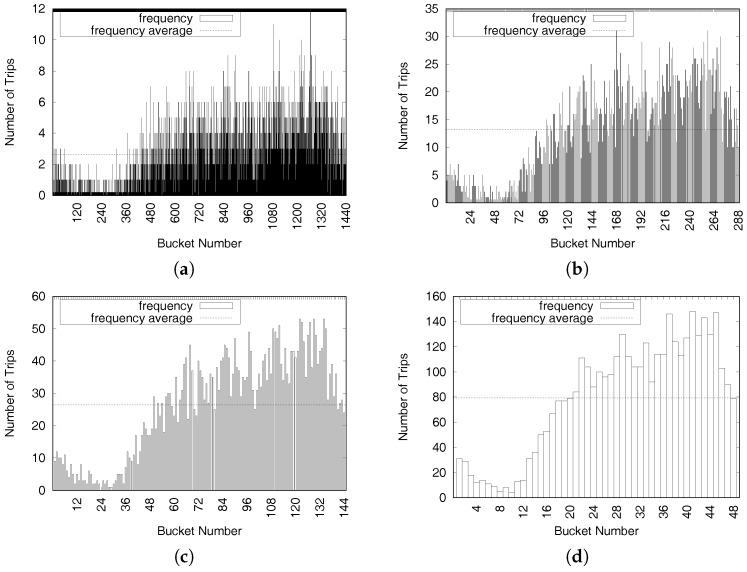
Distribution of taxi trip data. (**a**) Trip frequency per minute (average: 2.6); (**b**) Trip frequency per 5 min (average: 13.2); (**c**) Trip frequency per 10 min (average: 26.41); (**d**) Trip frequency per 30 min (average: 79.2).

Figure 5 depicts the distributions of New York taxi trip data in one specific area on Fridays during September 2015. When the size of a time period is set to one minute, as shown in Figure 5a, the total number of time periods (bucket number) is 1440 (=60/h × 24 h). The average value of trips (${\bar{x}}_{n_{1}}$) per time period is 2.6, and the maximum number of taxi trips in a time period is only 12. When the size of a time period is 30 min, as shown in Figure 5d, the total number of time periods is 48 (=2/h × 24 h), and the average value of trips per time period (${\bar{x}}_{n_{30}}$) is 79.2. Another consideration when deciding the size of a time period is average travel time from taxi trip dataset sample ${\bar{x}}_{tt}$. The average taxi driver finishes a trip in 14 min, according to the NewYork taxi trip dataset. Thus, we should set a reasonable value to the size of a time period.

Equation (1) explains how to determine the size of a time period (=time interval) by simultaneously considering two features such as the average taxi trip frequency during the time period and the average travel time for a taxi trip data set. In other words, the size is set to the minimum value of *i* that satisfies two conditions: (1) the average value of trips (${\bar{x}}_{n_{i}}$) should be larger than the multiplication of a number of candidate areas, $n_{PA}$, by a frequency $n_{freq}$; and (2) it should be smaller than the average travel time of taxi trips (${\bar{x}}_{tt}$).

(1) $$size_{tp}=\underset{i}{arg min}\;\left(\left\{i|\;\left({\bar{x}}_{n_{i}}>n_{PA}\times n_{freq}\right)\;\&\;\left(n_{i}<{\bar{x}}_{tt}\right)\right\}\right)$$

**Example** **3.** We established 10 min as the interval value of a time period for the New York taxi trip dataset, and 15 min for the Chicago dataset. Time periods in the New York dataset at 10 AM are maintained at 6 intervals such as [10:01, 10:10], [10:11, 10:20], [10:21, 10:30], [10:31, 10:40], [10:41, 10:50], and [10:51, 10:60]. Time periods of the Chicago dataset at 10 AM are maintained at 4 intervals, such as [10:01, 10:15], [10:16, 10:30], [10:31, 10:45], and [10:46, 10:60].

The time period is a basic unit of *DISPAQ* for retrieving profitable areas. For example, if a taxi driver specifies a query at 09:57 in New York City, it belongs to time period [09:51, 10:00]. Then, *DISPAQ* provides several profitable areas using 10-minute intervals, which can be computed from the current time.

#### 4.1.2. Area Summary

Since an extended route summary is a combination of an area summary and a route summary, we shall provide detailed explanations for these summaries. We begin with an intuitive observation. Taxi drivers plan their own routes after dropping off a passenger. They would like to select an area that guarantees high average fares and high passenger demand with a short waiting time. Their decisions for making high profits depends on area and time. The driver may know some candidate areas from his/her previous experience with the current location at a current time. Then, they might estimate taxi-passenger demand in candidate areas. Finally, they decide on one area for high profits according to past experiences.

To resemble a taxi driver’s decision process, a PQ-index needs two pieces of summary information. An area summary maintains all candidate areas that are computed from raw taxi trip data. For quickly identifying candidate profitable areas, we computed values with all combinations of (area, time) pairs. The PQ-index also utilizes the pair (area, time) as a spatio-temporal hash key.

Based on the above observation, we formally define an area summary as follows.

**Definition** **4.** *(Area Summary) An area summary* $AS_{ar,tp}$ *is represented as a 3-tuple* $\left(\mu_{f},L,pd\right)$*, where* ar *is an input area and* tp *is a time period. For the given area and time period, three values are computed from the raw taxi trip dataset: (1)* $\mu_{f}$ *as the average fare from area* ar *during time period* tp*, (2) L as a list of pickup probabilities in area* ar *at each time point during the time period* tp *(in other words, a list of candidate areas from area* ar *during the time interval* tp*), and (3)* pd *as passenger demand in area* ar *during time period* tp*.*

Given the formal definition for an area summary, we shall explain how to calculate the elements of the area summary. Note that a pair comprising area ar and a particular time tp is a spatio-temporal hash key for locating elements of an area summary.

Equation (2) explains how to compute the average fare from a taxi trip dataset. First, we compute a total sum of fares by summing up fare amount fa and tip amount tia from each taxi trip. Then, we divide this sum by the total number of taxi trips that start from area ar in time period tp.

(2) $$AS_{ar,tp}.\mu_{f}=\frac{\sum_{i=1}^{n_{ar,tp}}fa_{i}+ta_{i}}{n_{ar,tp}}$$

The second element of an area summary is a list of pickup probabilities at each time during the time period. We can obtain a pickup probability for each time point $t_{i}$ in time period tp as shown in Equation (3).

(3) $$AS_{A,tp}.L=\left\{\left(t_{i},\frac{n_{ar,t_{i}}}{n_{ar,tp}}\right)|t_{i}\in tp\right\}$$

**Figure 6:**
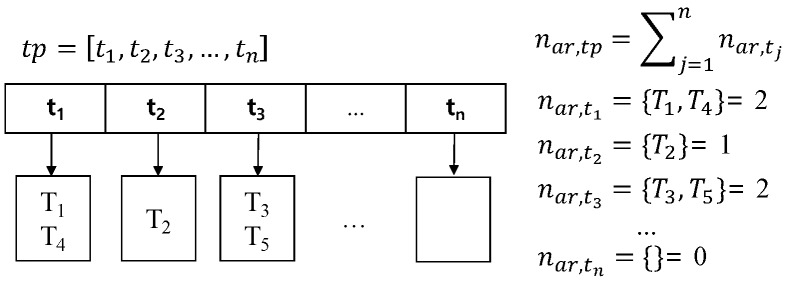
Computing pickup probabilities.

Figure 6 illustrates how to compute pickup probabilities. Assume that time period tp is an interval from $t_{1}$ to $t_{n}$. During time period tp, several trips could start from area ar. For example, two taxi trips $T_{1}$ and $T_{4}$ start at time point $t_{1}$, and taxi trip $T_{2}$ begins at time point $t_{2}$. We store the number of taxi trips for each time point $t_{i}$ into $n_{ar,t_{i}}$. The total number of trips during time period $n_{ar,tp}$ is a summation of all taxi trips $n_{ar,t_{i}}$. Each $t_{i}\in tp$ has a possibility to become the beginning time of a trip. Thus, we calculate the probability of each time point $t_{i}$ by dividing $n_{ar,t_{i}}$ by $n_{ar,tp}$.

Passenger demand is a probability defined in Equation (4). We can obtain this value by dividing the number of trips that started from ar in time period tp by the number of trips from all areas in time period tp.

(4) $$AS_{ar,tp}.pd=\frac{n_{ar,tp}}{n_{tp}}$$

**Figure 7:**
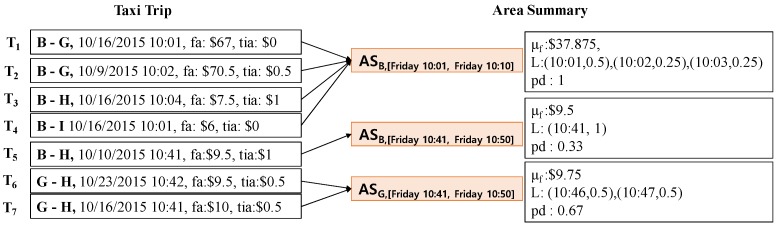
Computing an area summary.

**Example** **4.** *Figure 7 illustrates how to compute an area summary from a snippet of taxi trips. These taxi trips are the same dataset from Table 2. Consider the first four taxi trips:* $T_{1}-T_{4}$*. The pair (area* (B)*, time period [Friday 10:01, Friday 10:10]) can be identified from the pickup areas and times of taxi trips* $T_{1},T_{2},T_{3}$ *and* $T_{4}$*. Then, we can obtain area summary* $AS_{B,[Friday\;10:01,Friday\;10:10]}$ *by using the above equations and taxi trips* $T_{1},T_{2},T_{3}$ *and* $T_{4}$ *as follows:*

- $AS_{B,[Friday\;10:01,Friday\;10:10]}.\mu_{f}$ *= ((67 + 0) + (70.5 + 0.5) + (7.5 + 1) + (6 + 0))/4 = $37.875*
- $AS_{B,[Friday\;10:01,Friday\;10:10]}.L$ *= {(10:01,* $\frac{\left\{T_{1},T_{4}\right\}}{4}$*), (10:02,* $\frac{\left\{T_{2}\right\}}{4}$*), (10:03,* $\frac{\left\{T_{3}\right\}}{4}$*)}*
  = {(10:01, 0.5), (10:02, 0.25), (10:03, 0.25)}
- $AS_{B,[Friday\;10:01,Friday\;10:10]}.pd$ *= 4/4 = 1*

*In the same way, we can compute the two other area summaries* $AS_{B,[Friday\;10:41,Friday\;10:50]}$*,* $AS_{G,[Friday\;10:41,Friday\;10:50]}$ *by using taxi trips* $T_{5},T_{6}$ *and* $T_{7}$*.*

#### 4.1.3. Route Summary Calculation

Since the taxi trip dataset includes millions of routes, there exist several routes that have the same pickup area and drop-off area. These repeated routes can be summarized to provide valuable information when deciding on profitable areas. This leads us the following definition for a route summary.

**Definition** **5.** *(Route Summary) A route summary* $RS_{rt,tp}$ *is denoted by a 3-tuple* $\left(\mu_{d},\mu_{tt},\mu_{c}\right)$*, where* rt *is a route from area* $ar_{o}$ *to area* $ar_{d}$ *(Definition 3), and* tp *is a time period for computing aggregates. For the given route and time period, we compute three aggregated values: (1)* $\mu_{d}$ *as an average distance of a trip from* $ar_{o}$ *to* $ar_{d}$*, (2)* $\mu_{tt}$ *as an average travel time from* $ar_{o}$ *to* $ar_{d}$*, and (3)* $\mu_{c}$ *as the average expense a taxi driver incurs while driving from* $ar_{o}$ *to* $ar_{d}$*.*

Based on Definition 5, we calculate elements of a route summary from repeated taxi trips. Note that route rt and time period tp play a key role in identifying a route summary. The average distance is calculated with Equation (5). We compute the total sum of trip distances from the repeated routes and divide it by the number of routes ($n_{rt,tp}$).

(5) $$RS_{rt,tp}.\mu_{d}=\frac{\sum_{i=1}^{n_{rt,tp}}d_{i}}{n_{rt,tp}}$$

The average travel time can be computed with Equation (6). For each taxi trip *i*, we first compute travel time by subtracting pickup time $t_{p_{i}}$ from drop-off time $t_{d_{i}}$. The total travel time is the summation of the travel time from each route rt during time period tp. Then, we divide the total travel time by the total number of routes ($n_{rt,tp}$) to obtain the average travel time.

(6) $$RS_{rt,tp}.\mu_{tt}=\frac{\sum_{i=1}^{n_{rt,tp}}\left(t_{d_{i}}-t_{p_{i}}\right)}{n_{tr,tp}}$$

An average expense is computed with Equation (7). Since taxi trip datasets we used do not include the fuel fees, we use a simple model that fuel fees is proportional to the distance. In Equation (7), fule is the cost of gas per kilo meter. Thus, we sum fuel fees and toll fees ($toa_{i}$) of each route rt during time period tp. Then, we divide the total sum by the number of routes ($n_{rt,tp}$).

(7) $$RS_{rt,tp}.\mu_{c}=\frac{\sum_{i=1}^{n_{rt,tp}}toa_{i}+\left(d_{i}*fuel\right)}{n_{rt,tp}}$$

**Figure 8:**
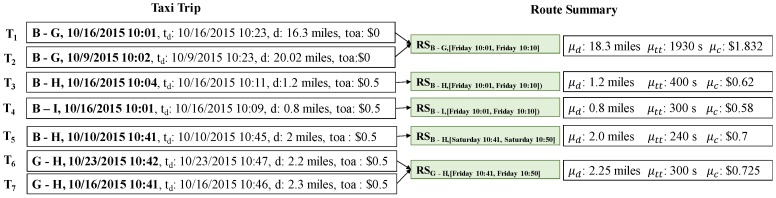
Route summary.

**Example** **5.** *Figure 8 illustrates how to compute an area summary from a snippet of taxi trips. From taxi trips* $T_{1}$ *and* $T_{2}$*, we can identify route* (B,G) *and time period [Friday 10:01, Friday 10:10].*

*A route summary for* $T_{1}$ *and* $T_{2}$ *can be calculated as follows:*

- $RS_{B-G,[Friday\;10:01,Friday\;10:10]}.\mu_{d}$ *= ((16.63 + 20.02)/2 = 18.325 miles*
- $RS_{B-G,[Friday\;10:01,Friday\;10:10]}.\mu_{tt}$ *= (2214 + 1654)/2 = 1934 s*
- $RS_{B-G,[Friday\;10:01,Friday\;10:10]}.\mu_{c}$ *= ((0 + 1.663) + (0 + 2.002))/2 = $1.8325*

*In the same way, we can calculate route summary* $RS_{C-H,[Friday\;10:41,Friday\;10:50]}$ *by using two trips* $T_{6}$ *and* $T_{7}$*. Trips* $T_{3}$*,* $T_{4}$ *and* $T_{5}$ *represent only one trip for each route; thus, aggregated values of route summaries are copied from each trip.*

#### 4.1.4. Extended Route Summary

**Figure 9:**
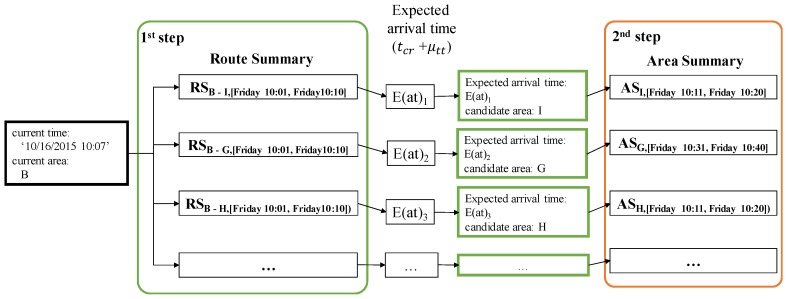
Two steps for constructing a profitable area map.

If an area summary and a route summary are managed and stored separately, we need to access these summaries in two steps to retrieve candidate profitable areas, as depicted in Figure 9. When a user provides a current area and a current time to our system, *DISPAQ* first checks route summaries that start from the user-specified area. Next, it estimates an expected arrival time and a candidate area from each route summary. Then, it searches area summaries to obtain benefits and expenses of the candidate area by using the pair (candidate area, expected arrival time) as a spatio-temporal key.

**Figure 10:**
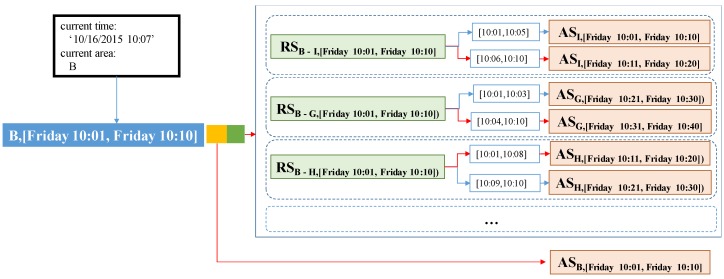
An extend route summary.

To fetch candidate area information in one step, we propose an extended route summary which is a combination of area summary and route summary. Abstractly, a route summary is augmented with area summaries that are retrieved with the pair (drop-off area of a route, expected arrival time period). Figure 10 presents an example of an extended route summary. A spatial temporal hash key has two elements denoted as a green box and an orange box. A green box is a pointer a set of extended route summaries. Each extended route summary has two pointers to the minimum and maximum time periods. A destination area summary is connected to each time period. An orange box is a pointer to an area summary which contains aggregated taxi trip information of the origin area. Each dashed rectangle means an extended route summary that is a combination of a route summary and area summaries.

Formally, we define an extended route summary as follows.

**Definition** **6.** (Extended Route Summary)

*An extended route summary* $ERS_{rt,tp}$ *contains a 5-tuple (*$RS_{rt,tp}$*,* $ti_{min}$*,* $AS_{ar_{d},ti_{min}}$*,* $ti_{max}$*,* $AS_{ar_{d},ti_{max}}$*), where* rt *is a route starting from area* $ar_{o}$ *to area* $ar_{d}$ *and* tp *is a time period. For the given route* rt *and time period* tp*, we calculate and maintain the following attributes as an extended route summary: (1)* $RS_{rt,tp}$ *is a route summary; (2)* $ti_{min}$ *is a time interval of the first partition for the expected arrival times; (3)* $AS_{ar_{d},tp_{min}}$ *is an area summary, where* $ar_{d}$ *is a destination area and* $tp_{min}$ *is a time period of the expected arrival times; (4)* $ti_{max}$ *is a a time interval for the second partition of the expected arrival times; (5)* $AS_{ar_{d},tp_{max}}$ *is a destination area summary, where* $ar_{d}$ *is a destination area and* $tp_{max}$ *is a time period of the expected arrival times.*

To augment a route summary with area summaries, we first need to compute the expected arrival times and decide the time period(s) for the expected arrival times. Since each route summary is associated with time period tp containing time points $\left[t_{1},t_{2},\ldots ,t_{n}\right]$, we compute the expected arrival time by adding average travel time $\mu_{tt}$ of the route summary to each time point $t_{i}\;\in \;tp$. The expected arrival time is used as a time period for the augmented (destination) area summary. Since we use a time period with a specific length computed with Equation (1), we should consider non-split and split cases when we add an area summary to a route summary. The non-split case happens when the range of expected arrival times is fully included within a specific time period. In this case, since there exists only one time period, we just add an area summary into the route summary of this time period. Otherwise, we split and map the range of expected arrival times into two time periods. The first time period is denoted as $tp_{min}$ and the second time period is denoted as $tp_{max}$. For each time period, we connected the area summary to the route summary.

**Figure 11:**
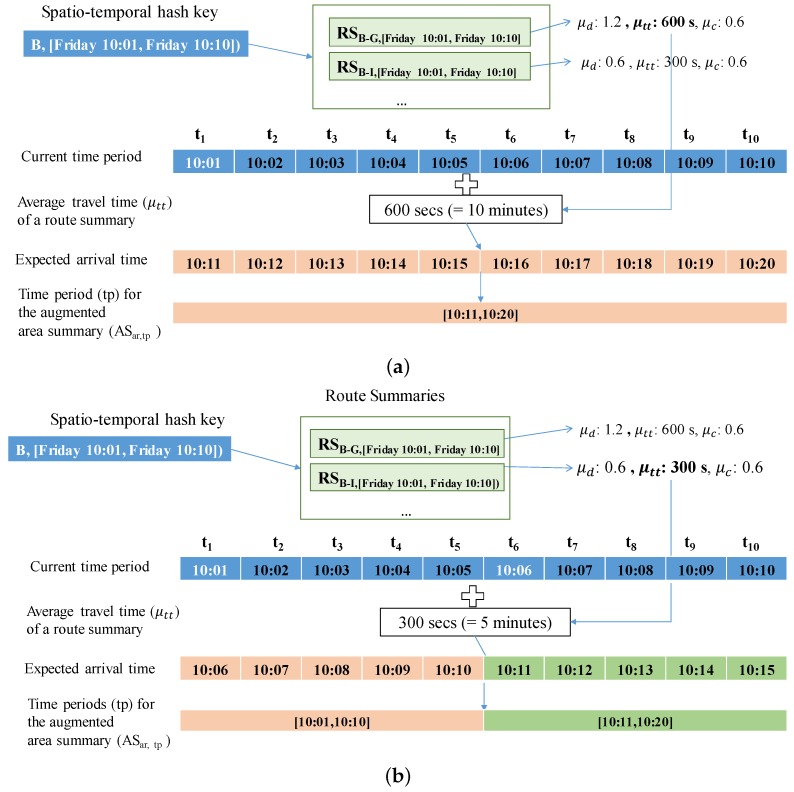
Arrival time mapping. (**a**) a non-split case; (**b**) a split case.

**Example** **6.** Figure 11 depicts how to compute the time period(s) for the expected arrival time. Assume that we are given area B and time period [Friday 10:01, Friday 10:10]. By using a pair (B, [Friday 10:01, Friday 10:10]), we can retrieve several route summaries that start from area B.

*First, consider a non-split case as shown in Figure 11a. In this case, we access route summary* $RS_{B-G,[Friday\;10:01,\;Friday\;10:10])}$*. For each time point of the time period [10:01, 10:10], we will add 10 min (600 s) which are obtained from the average travel time (*$\mu_{tt}$*) of the route summary. The expected arrival times are computed as 10:11, 10:12, ⋯, 10:20 and the time period of the expected arrival times is [10:11, 10:20]. This time period is fully included in the time period used in DISPAQ, and we do not need to split this time period. This period [10:11, 10:20] is used for the (destination) area summary.*

*Next, consider a split case as shown in Figure 11b. In this case, we access route summary* $RS_{B-I,[Friday\;10:01,\;Friday\;10:10]}$*. For each time point of the time period [10:01, 10:10], we will add 5 min (300 s) of the average travel time (*$\mu_{tt}$*) from the route summary. The expected arrival times are computed as 10:06, 10:07, ⋯, 10:10, 10:11, ⋯, 10:15. Since the range of the expected arrival times is not fully included within a specific time period, we split this range into two time periods* $tp_{min}=\;$ *[10:01, 10:10] and* $tp_{max}=\;$*[10:11, 10:20].*

However, sometimes there arises an exceptional case where the area summary of a destination area is empty. This will happen if none of taxi trips start from the destination area during the time period of the expected arrival times. We remove this destination area from candidate profitable areas due to lack of information.

**Example** **7.** *Consider Figure 10 again. Route summary* $RS_{B-I,[Friday\;10:01,\;Friday\;10:10]}$ *contains two split time periods: [10:01, 10:05] and [10:06, 10:10]. These time periods are computed as described in Example 6. We connect area summaries* $AS_{I,[Friday\;10:01,\;Friday\;10:10]}$ *and* $AS_{I,[Friday\;10:11,\;Friday\;10:20]}$ *to the corresponding time period of the route summary. If a user specifies the current time as “10/16/2015 10:07", then DISPAQ utilizes the second area summary* $AS_{I,[Friday\;10:11,\;Friday\;10:20]}$ *because the current time belongs to the second time period [10:06, 10:10].*

#### 4.1.5. Overall Design of a PQ-Index

**Figure 12:**
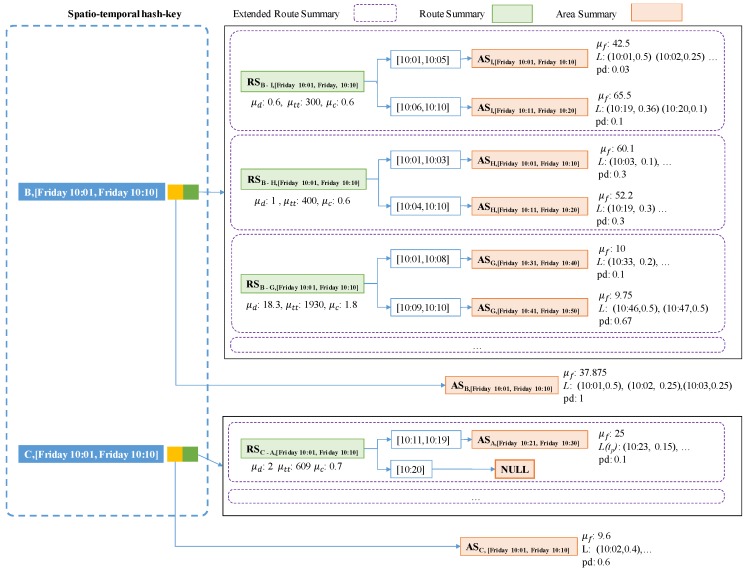
A PQ-index.

Figure 12 depicts an overall design of a PQ-index which exploits the concept of an extended route summary. In the left part, two pairs (B, [Friday 10:01, Friday 10:10]) and (C, [Friday 10:01, Friday 10:10]) play as spatio-temporal hash keys for the PQ-index. Each hash key is connected to an area summary and a set of extended route summaries.

The left pointer of the first hash key is used to visit an area summary that is represented as a yellow box. Area *B* has the value $18.40 as an average fare, a value of 0.2 as a passenger-demand probability during 10:01–10:10 on Friday, a set of pickup probabilities (10:05, 0.3), (10:06, 0.2), etc. By following the right pointer of the first hash key, we can obtain a set of extended route summaries. Repeated routes are aggregated as route summaries represented as green rectangles. The candidate profitable areas can be effectively retrieved by accessing area summaries that are connected to the route summaries.

### 4.2. Distributed PQ-Index Construction

In this subsection, we shall explain how to construct a PQ-index from raw taxi trip data that corresponds to the definitions in Section 4.1.

**Figure 13:**
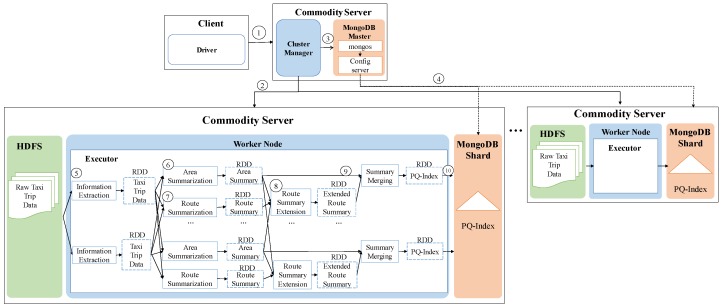
Distributed construction of a PQ-index.

To handle huge volumes of taxi trips efficiently, we devised a distributed PQ-index construction. Figure 13 illustrates the overview of PQ-index construction of our *DISPAQ* system, which is implemented on top of Spark. *DISPAQ* starts the construction process when a driver application in a client sends a command to a cluster manger (master) of Spark (①). The Spark cluster manager sends a configuration to all commodity servers that will function as worker nodes (②) or a MongoDB master (③). Note that we simultaneously use one commodity server as a Spark master and a MongoDB master for this configuration. These masters can be installed in different commodity servers. Worker nodes will process all of the distributed PQ-index construction steps, whereas the MongoDB master prepares shard servers for storage of the PQ-index as the final result of a worker node job (④). Again, the same commodity servers of Spark will serve as MongoDB shards (nodes).

Algorithm 1 explains the detailed steps executed in the commodity servers of Spark. A worker node executor reads huge volumes of taxi trip data stored in HDFS and extracts taxi trip information TI (Line 1 and denoted as ⑤). Note that circled numbers are illustrated in Figure 13. During the extraction process, the executor initializes the summary data structures and remove unused attributes. Then, the executor continues to group taxi trip information based on the pair (pickup area, time period) for the area summary (Line 4) and based on the pair (route, time period) for the route summary (Line 5). After grouping, the executor computes all possible combinations of the area summary and route summary: Lines 8 and 9, also denoted as ⑥ and ⑦. The extended route summary is built by connecting the area summary and route summary: Line 10 and ⑧. Then, a distributed PQ-index construction is completed by merging the area summary and the extended route summary that has the identical key: Line 11 and ⑨. Finally, the executor sends a constructed PQ-index to MongoDB shards (Line 12 and ⑩).

**Algorithm 1:** Distributed PQ-index Construction 
**Input**: Set of taxi trips *T* **Output**: PQ-index PQI // information extraction **  1:** Taxi trip information TI←informationExtraction(*T*); // grouping by area or route
**  2:** Initialize AG as a tuple of (pair (ar,tp), a list of taxi trip information); **  3:** Initialize RG as a tuple of (pair (rt,tp), a list of taxi trip information);
**  4:**
AG ← groupByAreaTP(TI); **  5:**
RG ← groupByRouteTP(TI); // construct basic summaries **  6:** Initialize ASP for an area summary; **  7:** Initialize RSP for a route summary; **  8:**
ASP ← BuildAreaSummary(AG); // Algorithm 2 **  9:**
RSP ← BuildRouteSummary(RG); // Algorithm 3 // PQ-index construction **10:** An extended route summary ERSP ← BuildExtendedRouteSummary(RSP); // Algorithm 4 **11:**
PQI ← mergeByKey(ASP,ERSP); **12:** return PQI;

Algorithm 2 depicts how to build an area summary. As explained in Section 4.1.2, this algorithm computes the summarized values of an area by applying Equations (2)–(4). Then, it creates the pair (spatio-temporal hash-key, constructed area summary) as output. In other words, this algorithm generates area summary $AS_{ar,tp}$ because $AG_{key}$ is represented as the pair (area, time period). 

**Algorithm 2:** Build an Area Summary 
**Input**: AG: a tuple (key, *L*), where key is a pair (area, time period) and *L* is a list of taxi information  **Output**: ASP: a pair (spatio-temporal hash-key key, an area summary AS) **1** Initialize AS as Area Summary; // calculate area summary value **2**
$AS_{key}$ ← $AG_{key}$; **3**
$AS_{key}.\mu_{f}$ is calculated from each group of $AG_{key,L}$; // Equation (2) **4**
$AS_{key}.List$ is computed from each group of $AG_{key,L}$; // Equation (3) **5**
$AS_{key}.pd$ is calculated from each group of $AG_{key,L}$; // Equation (4) **6**
ASP ← pair($AG_{key}$,AS); **7** return ASP;

Algorithm 3 presents steps for building a route summary. Basically, it implements Equations (5)–(7) to calculate elements of a route summary (Lines 3–5). This algorithm not only calculates the elements of a route summary but also computes the time intervals by considering split and non-split cases explained in Figure 11 (Line 8). Then, it returns RSP for easier construction of the extended route summaries. 

**Algorithm 3:** Build a Route Summary 
**Input**: RG: a tuple (key, *L*), where key is the pair (route, time period) key and *L* is a list of taxi information **Output**: RSP: a tuple (a pair (ar, tp), area, first time period, second time period, a route summary)  **1** Initialize RS as a route summary; // compute elements of a route summary  **2**
$RS_{key}$ ← $RG_{key}$;  **3**
$RS_{key}.\mu_{d}$ is calculated from each group $RG_{key,L}$; // Equation (5)  **4**
$RS_{key}.\mu_{tt}$ is computed from $RG_{key,L}$; // Equation (6)  **5**
$RS_{key}.\mu_{c}$ is calculated from $RG_{key,L}$; // Equation (7)  **6** a destination area $ar_{d}$ ←$RG_{key}.\mathrm{getDestArea}\left(\right)$;  **7** an origin area $ar_{o}$ ←$RG_{key}.\mathrm{getOriginArea}\left(\right)$; // compute two time intervals: $tp_{min}$
and
$tp_{max}$  **8**
ArrivalTimeMapping ($tp_{min}$, $tp_{max}$, $RS_{key}$); // make an RSP with time invtervals for the extension  **9** a spatio-temporal hashkey hkey ← a pair of ($ar_{o}$, $RG_{key}.tp$); **10**
RSP ← a tuple of (hkey, $ar_{d}$, $tp_{min}$, $tp_{max}$, RS); **11** return RSP;

Algorithm 4 illustrates the processes for building an extended route summary as explained in Section 4.1.4. We augment two area summaries for a given input route summary based on a destination area of the route and the expected arrival time period (Lines 3–6). 

**Algorithm 4:** Build an Extended Route Summary 
**Input**: RSP: tuple (key *k*, area ar, first time period $tp_{min}$, second time period $tp_{max}$, route summary rs) **Output**: ERSP: pair (spatio-temporal hash-key key, Extended Route Summary ERS) **1** Initialize ERS as Extended Route Summary; // Assign a route summary **2**
ERS.RS ← RSP.rs; **3**
$ERS.tp_{min}$ ← $RSP.tp_{min}$; **4**
$ERS.tp_{max}$ ← $RSP.tp_{max}$; // augmenting a route summary with area summries **5**
$ERS.AS_{min}$ ← GetAreaSummary
RSP.ar, $tp_{min}$; **6**
$ERS.AS_{max}$ ← GetAreaSummary
RSP.ar, $tp_{max}$; // combine a spatio-temporal hashkey with an extended route summary **7**
ERSP ← a pair of (key, ERS); **8** return ERSP;

### 4.3. Complexity Analysis of PQ-Index Construction

In this subsection, we analyze the complexity of a distributed PQ-index construction method by providing a serial execution cost and then a distributed execution cost. We use the cost model similar to that used for finding k-most promising products (k-MPP) [60].

To construct a PQ-index in a single commodity server, *DISPAQ* executes the several steps explained in Section 4.2. First, it extracts taxi trip information TI from a raw taxi trip dataset by removing unrelated data for profitable areas. Suppose, for given taxi trip dataset *D*, the time to extract the taxi trip information is $T_{ext}\left(D\right)$. We use |TI| to represent the size of the extracted taxi trip information. Next, *DISPAQ* generates area summaries and route summaries from TI and builds extended route summaries by augmenting a route summary with area summaries. The summary construction times are $T_{as}\left(|TI|\right)$ and $T_{rs}\left(|TI|\right)$. The total sizes of area summaries and route summaries are denoted by |AS| and RS|. Then, it combines the extended route summary and the area summary based on the pair (area, time period), which is a spatio-temporal hash-key. The extended route summary construction time is $T_{ers}\left(RS\right|+\left|TS|\right)$. The execution time of the merge step in Algorithm 1 is denoted as $T_{merge}$. Equation (8) represents the runtime complexity of constructing the PQ-index by summing up the sub-processes’ average runtime in a single commodity server:

(8) $$T_{PQ-index}^{S}=T_{ext}\left(D\right)+T_{as}\left(|TI|\right)+T_{rs}\left(|TI|\right)+T_{ers}\left(|RS\right|+\left|TS|\right)+T_{merge}$$

The runtime complexity of constructing a distributed PQ-index can be computed as follows. Assume that *N* commodity severs are used for the distributed construction and each server has a equally divided subset of data. We ignore the implementation overhead of synchronization and data communications among all servers. Equation (9) illustrates the complexity of distributed PQ-index construction in *N* commodity server environments.

(9) $$T_{PQ-index}^{D}=\frac{T_{ext}\left(D\right)+T_{as}\left(|TI|\right)+T_{rs}\left(|TI|\right)+T_{ers}\left(RS\right|+\left|TS|\right)+T_{merge}}{N}$$

## 5. Processing Profitable-Area Query

In this section, we shall explain how to find profitable areas when a user query is given to *DISPAQ*. The processing of a profitable-area query is executed in two steps: (1) retrieve candidate profitable areas into a profitability map by utilizing the PQ-index and (2) refine candidate profitable areas in the profitability map by exploiting extended skyline query processing.

### 5.1. Profitable-Area Query

As explained in Section 1, several factors affect taxi drivers’ strategies to determine profitable areas that guarantee more passengers. Since our *DISPAQ* system solves this problem based on skyline query processing, we define three major terms under the concept of skyline query processing.

To formulate a profitable-area query, we begin by defining a profitable area.

**Definition** **7.** (Profitable Area)

*A profitable area* $PA_{ar,tp}$ *is defined by a 4-tuple* $\left(p,pd,t_{cr},d_{cr}\right)$*. The input parameters* ar *and* tp *mean area and time period, respectively. The profitable area* $PA_{ar,tp}$ *contains four aggregated values: (1) p as profit, (2)* pd *as passenger demand, (3)* $t_{cr}$ *as cruising time, and (4)* $d_{cr}$ *as cruising distance.*

Profitable area $PA_{ar,tp}$ contains the aggregated values of an area that follows Definition 2 and is denoted by a geohash value. Several factors affect taxi drivers’ passenger search strategies. Thus, we chose four factors from the taxi trip data explained in Section 3.2. The aggregated values of these factors are calculated based on an area ar and a time period tp since these values vary with each pair (area, time period); *p* denotes an approximate amount of income for taxi drivers if they pick up passengers from area ar at time period tp and pd denotes the probability of a taxi driver taking passengers from area ar compared with other areas within the same time period, tp; $t_{cr}$ is the average elapsed time it takes taxi drivers to get passengers in area ar from the current area of the input query; $d_{cr}$ means a distance between the area ar from the current area of the input query. How to compute these four values is explained in Section 5.

A profitability map $PM_{ar,tp}$ is a set of profitable areas. After *DISPAQ* receives the current location and current time from a user, it computes a profitability map that contains candidate profitable areas from the pair (current location, current time).

**Definition** **8.** *(Profitability map) Profitability map* $PM_{ar,tp}$ *is a set of profitable areas* [PA[1],PA[2],..,PA[n]]*, where* PA[i] *is a profitable area in the form of* $PA_{ar,tp}$ *in Definition 7,* ar *means an area and* tp *denotes a time period.*

Assume that dataset *D* consists of profitable areas. In other words, D={PA[1],PA[2],..,PA[n]}. Then, dataset *D* follows the definition of profitability map PM. As we can see, profitable area PA[i] can include several factors and values, where each factor serves as one coordinate (dimension) of PA[i]. Thus, if we consider four factors, then the dimension of PA[i] is 4. Profitable area PA[i] is not dominated if it is as good or better in all dimensions and better in at least one dimension. If PA[i] dominates $PA_{j}$, we represent it as PA[i]≺PA[j]. The skyline of PM, represented by SL(PM), is a subset of PM where every profitable area in SL(PM) is not dominated by every other point in PM [21].

**Figure 14:**
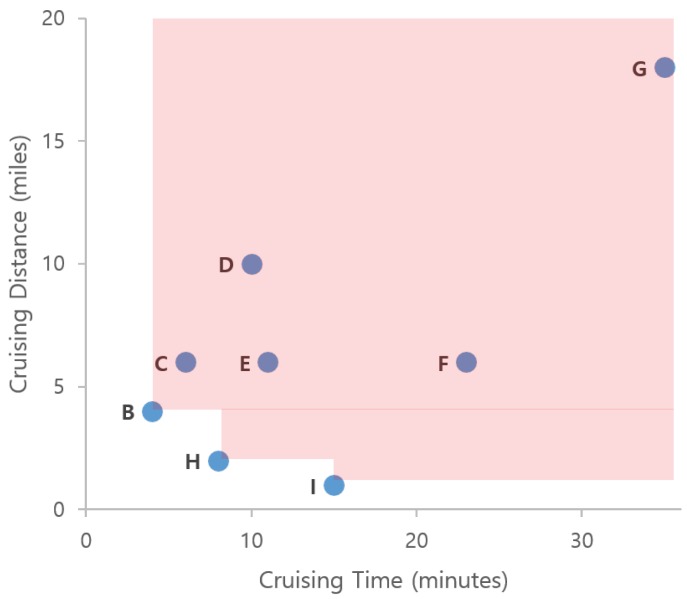
Skyline.

A visualization example of skyline from taxi trip data is illustrated in Figure 14. To simplify the problem, we only consider two factors (dimensions) from the table in Figure 1b for deciding the skyline of profitable areas. When we read the first row in the table, area *B* is considered a skyline because we do not have other areas for comparison. Then, we read area *C* and find that *C* is dominated by area *B* because it has a longer cruising time and a longer distance. This condition also occurs in areas *D*, *E*, *F*, and *G*. Next, when we read area *H*, we regard area *H* as an element of a skyline because its distance is smaller although the cruising time is longer than area *B*. Then, we also consider area *I* as an element of a skyline, because this area dominates other skyline areas in the cruising distance factor. Finally, we decide areas *B*, *H*, and *I* are the skyline areas. Every time we read a taxi trip, we need to check the dominance of the trip against every other taxi trip by using all dimensions. By applying a dominance test, we can ensure that a profitable area is not dominated by other profitable areas.

Finally, a profitable-area query is defined as follows.

**Definition** **9.** *(Profitable-Area Query) Given a pair comprising current location and current time* (cl,ct)*, a profitable-area query selects non-dominated areas (skylines) from profitability map* $PM_{ar,tp}$ *which can be represented as* $SL(PM_{ar,tp})$*. Each profitable area,* PA[i]*, in* $SL(PM_{cl,ct})$ *satisfies the condition* $SL\left(PM_{ar,tp}\right)=\{PA\left[i\right]\in PM_{ar,tp}|∄PA\left[j\right]\left(\neq PA\left[i\right]\right)\in PM_{ar,tp}$ *s.t.* PA[j]≺PA[i]}

**Figure 15:**
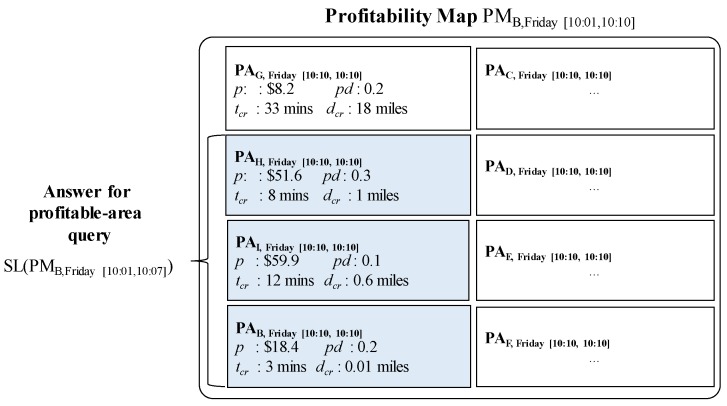
Profitable-area query data model.

**Example** **8.** Consider Figure 14 again. Assume that a user sends his location (B) and current time (2016/12/18 10:11) to DISPAQ. DISPAQ computes candidate profitable areas from taxi trip data depicted in Table 2 and creates a profitability map as shown in Figure 15. After executing the profitable-area query, it returns areas B, H and I as the results, based on Definition 9.

Figure 15 illustrates the relationships among the three terms: (1) profitable area, (2) profitability map and (3) the answers for profitable-area query processing. How to construct and utilize the profitable map will be explained in the following subsection.

### 5.2. Retrieving Candidate Profitable Areas into a Profitability Map

After constructing the PQ-index, *DISPAQ* is ready to receive a user query that contains an area from the current location and a time period from the current time. A pair (area, time period) helps *DISPAQ* to efficiently retrieve candidate areas by exploiting the extended route summaries of the PQ-index. When *DISPAQ* builds a PQ-index, it pre-computes benefits of candidate areas by considering several factors and stores them in the extended route summaries. A set of candidate profitable areas is collected into a profitability map in our *DISPAQ* system. Note that the formal definitions of a profitable area and a profitability map are defined in Definition 7 and Definition 8, respectively.

Consider again Figure 15, which illustrates an example profitable map including several candidate profitable areas. Each profitable area maintains four factors (profit, passenger demand, cruising time, and cruising distance) as attributes. As explained in Section 4.1, these factors can easily be accessed by exploiting the PQ-index.

Assume that current area ca and time period tp are used for retrieving candidate profitable areas into profitability map $PM_{ca,tp}$. The route summary provides average trip distance $RS_{rt,tp}.\mu_{d}$ which becomes the cruising distance of profitable area $PA_{ar_{d},tp}.d_{cr}$. Route rt can be selected when it starts from area ca. In other words, the origin area of the route, $rt.ar_{o}$, is the current area ca. Candidate profitable area $ar_{d}$ of route rt is extracted from the destination area of route $rt.ar_{d}$. An area summary gives passenger-demand probability $AS_{ar_{d},tp}.pd$ which will be $PA_{ar_{d},tp}.pd$. Profit $PA_{ar_{d},tp}.p$ is the result of subtracting the average cost of route summary $RS_{rt,tp}.\mu_{c}$ from the average fare of area summary $AS_{ar_{d},tp}.\mu_{f}$, which is expressed in Equation (10):

(10) $$PA_{ar_{d},tp}.p=AS_{ar_{d},tp}.\mu_{f}-RS_{rt,tp}.\mu_{c}$$

**Figure 16:**
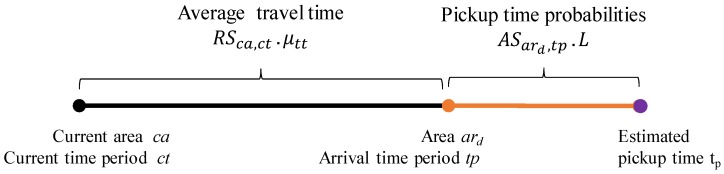
Cruising Time.

Since cruising time is the approximate time a taxi driver should take to get a new passenger, two values are needed to compute cruising time. Figure 16 depicts how to calculate cruising time. First, a driver takes arrival time $t_{a}$ to move from current area ca to candidate profitable area $ar_{d}$. We compute arrival time period tp by adding current time period ct to the average travel time of a route, $RS_{ca,ct}.\mu_{tt}$. Second, we estimated pickup time $t_{p}$ of candidate profitable area $ar_{d}$ by choosing a time point that has the maximum pickup probability in $AS_{ar_{d},tp}.L$. Then, we can obtain the cruising time in profitable area $PA_{ar_{d},tp}.t_{cr}$ by subtracting the current time from estimated pickup time $t_{p}$.

**Figure 17:**
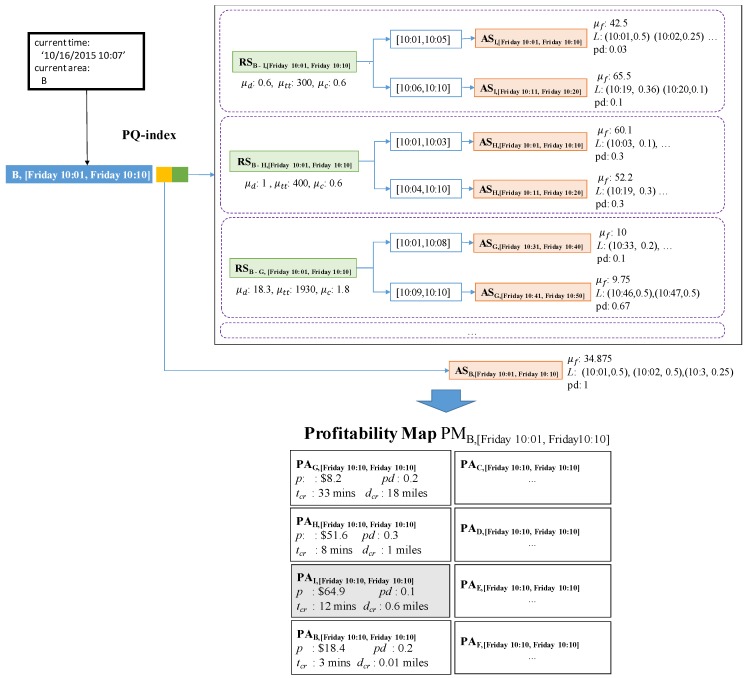
How to retrieve a profitability map.

**Example** **9.** *Figure 17 illustrates how to retrieve candidate profitable areas and store them in a profitability map. DISPAQ receives a user query specified by current area B and current time 10/16/2015 10:07. The current time will be immediately changed into the current time period it belongs to. The pair (B, [Friday 10:01, Friday 10:10]) works as a spatio-temporal hash key of the PQ-index shown in the top of Figure 17. Three route summaries are selected:* $RS_{B-I,[Friday\;10:01,\;Friday\;10:10]}$*,* $RS_{B-H,[Friday\;10:01,\;Friday\;10:10]}$ *and* $RS_{B-G,[Friday\;10:01,\;Friday\;10:10]}$*. Because they start from B on Friday between 10:01–10:10. Drop-off areas I, H and G of these routes become candidate profitable areas, which will be included in the profitability map.*

*Let us consider the first area I. For area I, we choose area summary* $AS_{I,Friday\;10:11,\;Friday\;10:20}$ *because the current time is inside the range [10:06–10:10]. By combining* $RS_{B-I,[Friday\;10:01,\;Friday\;10:10]}$ *and* $AS_{I,[Friday\;10:11,\;Friday\;10:20]}$ *of the extended route summary, we can calculate elements of profitable area* $PA_{I,[Friday\;10:11,\;Friday\;10:20]}$ *as follows:*

- $PA_{I,[Friday\;10:01,\;Friday\;10:10]}.p=\$65.5-\$0.6=\$64.9$
- $PA_{I,[Friday\;10:01,\;Friday\;10:10]}.pd=0.1$
- $PA_{I,[Friday\;10:01,\;Friday\;10:10]}.t_{cr}=\underset{t}{\mathrm{argmax}}\left(\left\{\left(10:19,0.36\right),\left(10:20,0.1\right)\right\}\right)-10:07=12\;min$
- $PA_{I,[Friday\;10:01,\;Friday\;10:10]}.d_{cr}=0.6\;miles$

The third elements computed as follows. We can obtain the time which maximizes a list of pickup probabilities and then subtract the current time from it.The other profitable areas can take values of their attributes in the same manner.

### 5.3. Refining Candidate Profitable Areas

A profitability map maintains a set of candidate profitable areas. However, all areas included in the profitability map cannot be recommended to a taxi driver who sends a query to the system. A refinement step is to remove non-dominated profitable areas from the profitability map based on the concept of skyline query processing. For this purpose, we suggest a Z-skyline method, which is extended skyline processing with a Z-order filling curve.

#### 5.3.1. Z-Order Values to Profitable Areas

As explained in Section 5.1, skyline query processing facilitates refining candidate profitable areas in a profitability map. However, computing skylines from a whole dataset is an expensive operation since it requires comparison of each element to all the other elements in the dataset which is called a dominance test. Thus, to reduce expensive dominance tests, Z-order space filling curve is utilized for computing skylines [25]. *DISPAQ* adopts skyline processing with Z-order, called Z-Skyline, as a basic algorithm for refining candidate profitable areas.

A Z-order curve accommodates multidimensional data into one dimensional data, called z-values. Z-values are computed from interleaving the bits of dimensional positions. We can extract a dimensional position value for each dimension (factor) in a profitable area. The dimensional position can be defined as follows.

**Definition** **10.** *(Dimensional Position) Let* $DP(PA_{d_{i}})$ *denote a dimensional position for dimension* $d_{i}$ *of a profitable area* PA*. Then,* $DP(PA_{d_{i}})$ *is defined as* $\frac{PA_{d_{i}}}{\left\lceil \frac{max(\left\{PA_{j}d_{i}|PA_{j}\in PM\right\})}{k}\right\rceil }$*, where* $d_{i}$ *is a factor (attribute) of* PA *and k is the number of partitions for* $d_{i}$*.*

**Figure 18:**
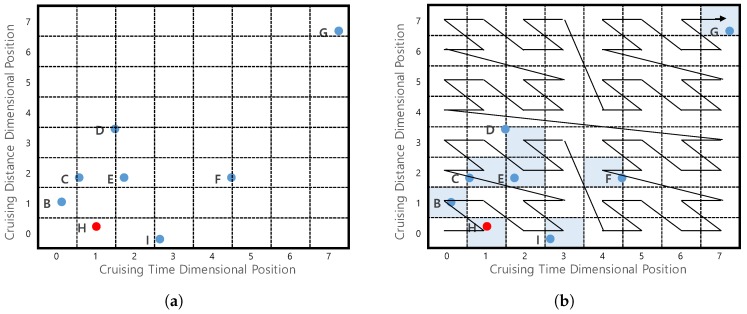
Computing Z-order values. (**a**) Dimensional positions; (**b**) Z-order values.

**Example** **10.** Consider the example in Figure 18a which shows profitable areas from Figure 1b. To simplify the explanation, we only consider two factors (dimensions) for the profitable area. Assume that we divide a cruising time dimension and a cruising distance dimension into eight partitions. Since profitable area G has 7 as the cruising time dimensional position and 7 as the cruising distance dimensional position, we will use the notation G(7,7). In the same way, the dimensional positions for profitable areas H, I and B are represented as H(1,0), I(3,0) and B(0,1).

We can formally define a Z-order value of a profitable area based on the dimensional positions of attributes in the profitable area.

**Definition** **11.** *(Z-order Value) A Z-order value of a profitable area is defined as* $Z\left(PA\right)=bin(DP\left(PA_{d_{1}},\;\cdots ,\;DP\left(PA_{d_{j}}\right)\right)$*, where j is the number of factors (attributes) of profitable area, and* bin(·) *is a function to transform a decimal value into a binary value by applying bit-shuffling of all dimensions (attributes) from* $d_{1}$ *to* $d_{j}$*. The length of a binary digit is determined by the number of partitions k.*

**Example** **11.** *Consider Figure 18a, which depicts four profitable areas G, H, I, and B. We obtained dimensional positions of every factor in the profitable areas, such as G(7,7), H(1,0), I(3,0) and B(0,1). The three-digit binary representations are G(111,111), H(001,000), I(011,000), and B(000,001), because the number of partitions is 8*($2^{3}$*). The* bin(·) *function interleaves the binary representations of all factors. In our case, we use the y-axis digit first. Then, we can obtain Z-order values for four areas* Z(G) *= 11 11 11,* Z(H) *= 00 00 01,* Z(I) *= 00 01 01, and* Z(B) *= 00 00 10. These binary values correspond to 63, 1, 5, and 2 in decimal format. Note that the decimal Z-order values correspond to the orders shown in Figure 18b.*

Skyline query processing can be improved with two characteristics of a Z-order curve: (1) automatic clustering of the data and (2) monotonic order [25].

The first characteristic can be achieved if we consider the same prefixes of Z-order values for profitable areas. For example, profitable areas *H*, *B*, *C*, *E* and *I* could belong to one cluster because they have the same first two bits “00”. We call this cluster as a region.

Formally, a region can be defined as follows.

**Definition** **12.** *(Region) Region* $R_{i}$ *is a set of profitable areas that satisfies the following condition:* $R_{i}\;=\;\left\{PA_{j}\;|\;\forall PA_{j}\;\in \;R_{i},\;Z\left(PA_{j}\right)=i\right\}$*.*

**Figure 19:**
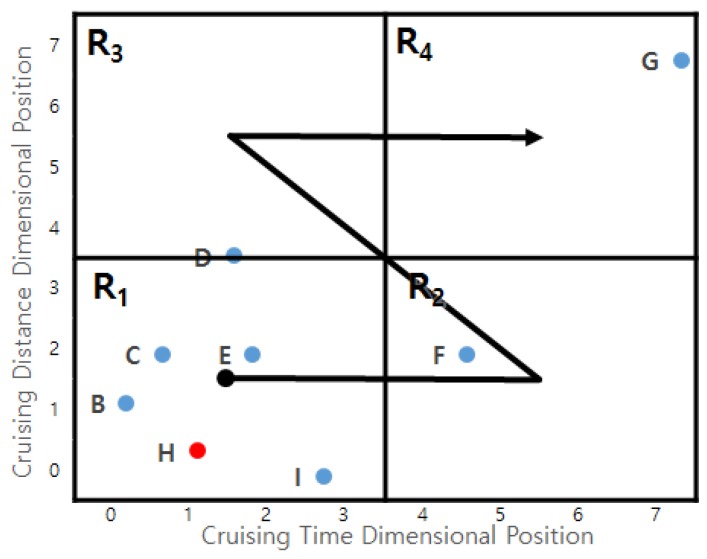
A region example.

**Example** **12.** *Figure 19 depicts a clustering example. Assume that we map profitable areas of two dimensions into four regions by considering the first two bits of Z-order values. Then, profitable areas B, H, C, E and I are clustered into Region* $R_{1}$*, whereas a profitable area G is clustered into Region* $R_{4}$*.*

The second characteristic of the Z-order curve (monotonic ordering of Z-order values) guarantees that a small dimensional position comes before a larger dimensional position. A profitable area with a small Z-order value is accessed before a profitable area with a large Z-order value, which means a dominating profitable area is accessed before the dominated profitable area. This removes unnecessary dominance tests and candidate re-examinations [25].

**Example** **13.** *Consider Figure 19 again. Region* $R_{1}$ *becomes the first accessed region, followed by Region* $R_{2}$*, Region* $R_{3}$*, and finally Region* $R_{4}$*. In region* $R_{1}$*, five profitable areas exists: B, C, E, H, and I. Among these regions, H is accessed first, followed by B, I, C and E during skyline query processing.*

Owing to two characteristics of the Z-order curve, our Z-skyline approach effectively minimizes the dominance test during the skyline process. Automatic clustering enables *DISPAQ* to utilize efficient block-based dominance tests, instead of checking the pairwise profitable area dominance test. Monotonic ordering prevents unnecessary candidate re-examinations. Thus, the distributed profitable-area query processing of *DISPAQ* is mainly based on the following Lemma [25].

**Lemma** **1.** *Given two regions,* $R_{i}$ *and* $R_{j}$*, the following three cases can happen during the refining process for final profitable areas.*

*(1)*  *All profitable areas in region* $R_{j}$ *are dominated by region* $R_{i}$*.*
*(2)*  *Some profitable areas in* $R_{j}$ *may be dominated by others in* $R_{i}$*.*
*(3)*  *All profitable areas in region* $R_{j}$ *are not dominated by region* $R_{i}$*.*

**Proof.**  We prove the lemma case by case. Let us denote a profitable area with a maximum Z-order value in $R_{i}$ ($R_{j}$) as $PA_{max}^{i}$ ($PA_{max}^{j}$) and a profitable area with a minimum Z-order value in $R_{i}$ ($R_{j}$) as $PA_{min}^{i}$ ($PA_{min}^{j}$).

**Figure 20:**
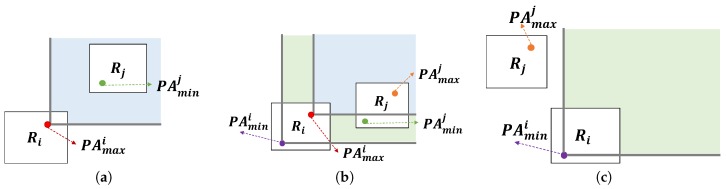
Three cases when refines candidate profitable areas. (**a**) case 1; (**b**) case 2; (**c**) case 3.

- Case 1: This happens when $PA_{max}^{i}$ dominates $PA_{min}^{j}$. Figure 20a depicts this case. Since the other profitable areas in $R_{i}$ dominate $PA_{max}^{i}$, they have smaller Z-order values. $PA_{min}^{j}$ also dominates the others in $R_{j}$ since it has the smallest Z-order value in $R_{j}$. Thus, any pairs of two profitable areas $PA_{k}\;\in \;R_{i}$ and $PA_{l}\;\in \;R_{j}$ satisfy the condition that $PA_{k}$ dominates $PA_{l}$. In other words, $R_{i}$ dominates $R_{j}$.
- Case 2: This happens when $PA_{max}^{i}$ does not dominate $PA_{min}^{j}$ and $PA_{min}^{i}$ dominates $PA_{max}^{j}$. In this case, profitable area $PA_{max}^{j}$ in $R_{j}$ is dominated by profitable area $PA_{min}^{i}$ in $R_{i}$. Thus, the case holds.
- Case 3: This happens when $PA_{min}^{i}$ does not dominate $PA_{max}^{j}$ as shown in Figure 20c. We will prove this case by contradiction. Assume profitable area $PA_{k}\;\in \;R_{i}$ dominates profitable area $PA_{l}\;\in \;R_{j}$. Then the z-oder value of $PA_{k}$ is smaller than that of $PA_{l}$. Since we choose profitable area $PA_{k}$ in $R_{i}$, the Z-order value of $PA_{k}$ is larger than that of $PA_{min}^{i}$. The Z-order value of $PA_{l}$ is smaller than that of $PA_{max}^{j}$. If we combine the above statements, we could conclude that Z-order value of $PA_{min}^{i}$ is smaller than that of $PA_{max}^{j}$. In other words, $PA_{min}^{i}$ dominates $PA_{max}^{j}$. This contradicts the case.

☐

#### 5.3.2. Profitable-Area Query by Z-Skyline Method

We apply a Z-skyline method to answer profitable-area queries on candidate areas included in a profitability map. As explained before, after receiving a user query, *DISPAQ* constructs a profitability map by exploiting the PQ-index. Then, it calculates z-values of candidate profitable areas in the profitability map. A small z-value means that the profitable area dominates with a high probability the other areas in all dimensions.

Algorithm 5 describes the proposed Z-skyline algorithm for answering a profitable-area query. It begins by initializing the final profitable results and a set of regions (Line 1 and Line 2). Z-order values of candidate profitable areas in the with a high probability map are computed in Line 3 and a set of regions, SR, is calculated in Line 4. Then, final profitable areas are obtained based on the three cases in Lemma 1 (Lines 5–17). In case 2, we merge two sets of profitable areas in FP and r.pal and again perform the dominance test. Note that we can skip the dominance test in the case 1.

To prove the correctness of Algorithm 5, we use the loop-invariant technique [61]. This approach examines the correctness of the algorithm in three loop stages: (1) initialization; (2) maintenance; and (3) termination. Thus, we can prove the correctness of the Z-skyline algorithm for refining profitable areas by following the loop-invariant verification method.

**Theorem** **1** (Correctness of the Z-skyline algorithm)**.** *The profitable-area query algorithm is correct with this loop invariant: for any step in a loop, the final profitable areas,* FP*, is a subset of non-dominated areas from a profitability map* PM*.*

**Proof.**  **Initialization:** Before an iteration is started, FP is initially empty. A set of regions SR consists of pairs (z-value, a list of profitable areas) which are areas constructed from PM by grouping profitable areas based on Z-order values.

**Maintenance:** For each iteration, after checking the emptiness of FP, the algorithm deals with three cases to determine whether profitable areas of a region *r* become a part of FP:

- When FP is empty: profitable areas of region *r* will added to FP by invoking the dominance test. Thus, FP contains non-dominated areas.
- When FP is not empty: Candidate profitable areas of region *r* should be handled based on the three cases in Lemma 1, which guarantees that only non-dominated areas will be added to FP. Thus, FP also contains a set of non-dominated areas in the case.

**Termination:** At the end of the iteration, FP contains a subset of non-dominated areas from profitability map PM.

**Correctness:** This loop-invariant method proves that the algorithm will be terminated and produce the correct results. ☐

**Algorithm 5:** Z-skyline for Refining Profitable Areas
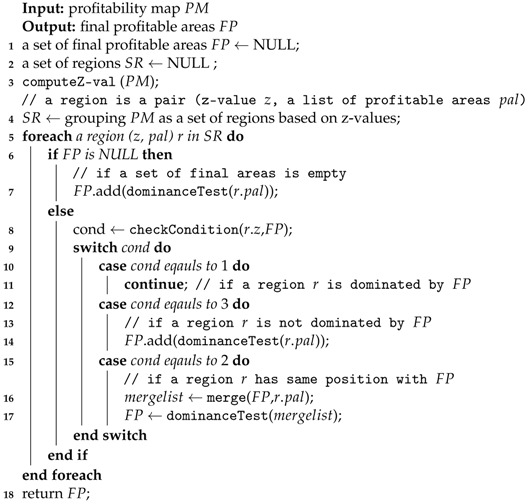

**Figure 21:**
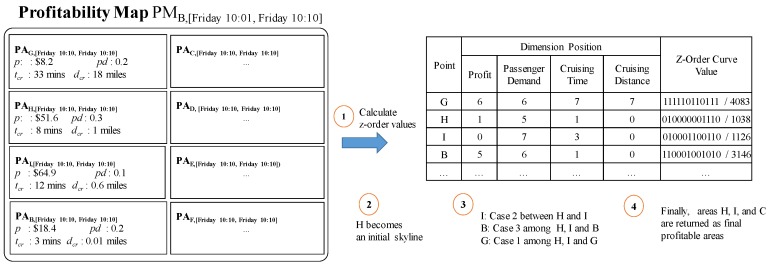
An example of profitable-area query processing.

**Example** **14.** *We will use candidate profitable areas from Figure 15 to illustrate the algorithm. Figure 21 depicts the steps in the Z-skyline algorithm by considering four factors (attributes) of a profitable area. First, Z-order computation is applied to candidate profitable areas included in* $PM_{B,[Friday\;10:01,\;Friday\;10:10]}$*. The Z-order value is presented in the table of Figure 21. Since area H has the smallest Z-order value, it will be accessed first and becomes an initial area skyline. Region* $r_{1038}$ *becomes a skyline region. Next, the algorithm continues to check conditions for each region. Area I (region* $r_{1126}$*) becomes the next accessed region. It is added to the skyline regions, being the second case in Lemma 1. Later, area B (region* $r_{3146}$*) is compared to the pre-computed skyline areas (areas I and H) and is included in the final answers. This is the third case in Lemma 1. Next, area G (region* $r_{4083}$*) is dominated by area H, and it will not be included in the final answers. After checking other profitable areas, profitable areas H, I and B are returned as final profitable areas.*

### 5.4. Distributed Profitable-Area Query Processing

#### 5.4.1. A Distributed Z-Skyline Approach

Dealing with the huge volumes of taxi trip data from major urban cities requires a scalable approach using several commodity servers. For this purpose, we implemented distributed profitable-area query processing on the top of the Apache Spark Core [22] which is a processing framework for distributed computing. Apache Spark supports a parallel processing by dividing the whole job into several sub-processes and merges separated intermediate results of the sub-processes. The distributed profitable-area query processing that utilizes the Z-skyline algorithm is divided into two steps: (1) a local Z-skyline and (2) a global Z-skyline. In the local Z-Skyline, all commodity servers of *DISPAQ* find local profitable areas via Z-Skyline which is explained in Algorithm 5. These intermediate local profitable areas need to be merged in one commodity server by the global Z-skyline computation. The results of the global Z-Skyline are the final profitable areas and will be returned to the user. Note that the global Z-skyline is implemented with Algorithm 5.

**Figure 22:**
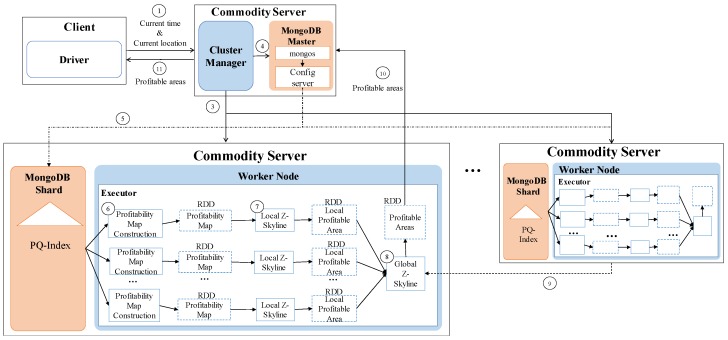
Distributed profitable-area query processing.

Figure 22 illustrates the distributed profitable-area query processing based on Spark. A client receives profitable-area query *q* from a user (①). The crucial part of the client is a driver that specifies the Spark configuration such as the transformations and actions on RDDs. The driver sends the configuration and query *q* to the Spark master(②). Then, the Spark master sends the Spark configuration to all worker nodes (③) and the parameters of query *q* to a MongoDB master (mongos) which is located in one of worker nodes (④) The MongoDB master sends query *q* to all shards (⑤). A MongoDB shard first obtains the part of the PQ-index corresponding to user query *q*, and divides the selected PQ-index in several RDDs (resilient distributed datasets), sending it to an executor of the same node for reducing data movement among worker nodes (⑥). A commodity server of *DISPAQ* computes a profitability map from the loaded PQ-index of the executor and removes dominated areas via local Z-Skyline (⑦). *DISPAQ* executes the global Z-Skyline to obtain final profitable areas from these local Z-skyline results (⑧ and ⑨). After completion of the global Z-Skyline processing, *DISPAQ* returns the final profitable areas as the results of query *q* (⑩ and ⑪).

#### 5.4.2. Optimizing a Distributed Z-Skyline Approach

The efficiency of the distributed profitable-area query processing depends on the local Z-skyline since the size of intermediate results influences the performance of the global Z-skyline. If a local Z-Skyline still retrieves a profitability map containing a lot of candidate profitable areas, the global Z-skyline might be the bottleneck of the whole process, because it needs to produce final profitable areas by merging the all intermediate results.

**Figure 23:**
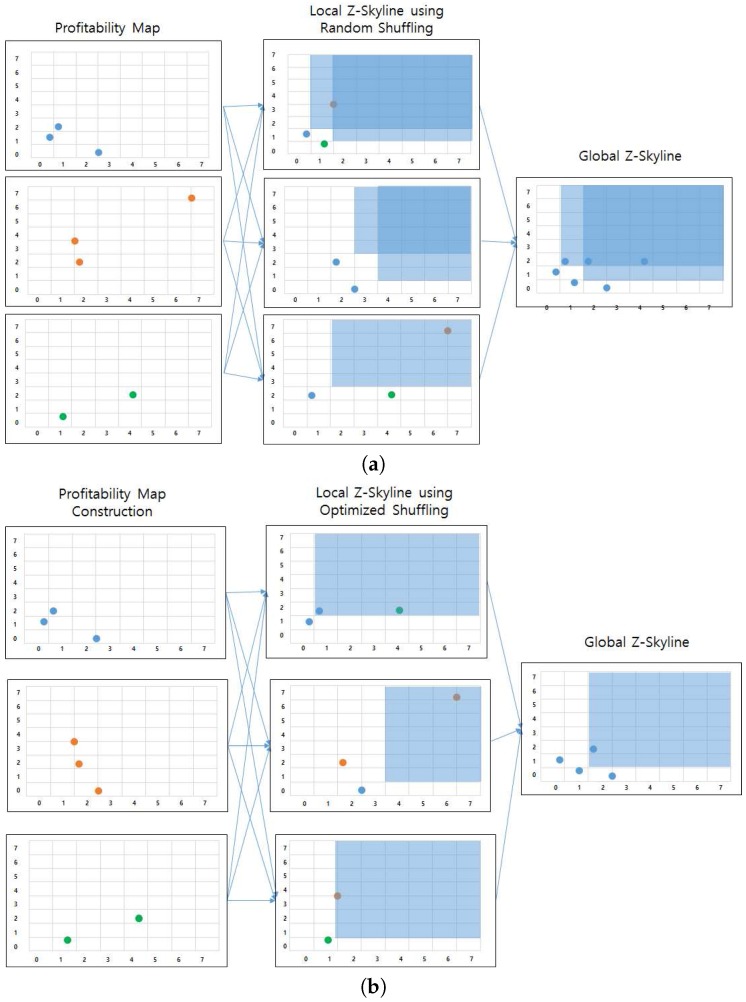
Data shuffling optimization. (**a**) Z-Skyline using random shuffling; (**b**) Z-Skyline using optimized shuffling.

At a local Z-skyline, each partition needs at least one killer area or region that removes most of the dominated areas. However, the intermediate profitability maps are built from random distribution of the profitable areas, which creates an unbalanced distribution of candidate profitable areas. This happens due to the default settings of Spark. Figure 23a depicts this case. The bottom partition has a profitability map containing a single killer region, whereas the top partition and the middle partition require two regions to eliminate all dominated region. In addition, the positions of killer regions are not as good as the position of the killer region in the bottom partition. Thus, the global Z-skyline must consider six candidate profitable areas to decide on the final profitable areas.

To optimize a local Z-skyline process, we propose an optimized shuffling method that avoids unbalanced profitable area distribution. As we explained in Section 5.3.1, the Z-Skyline has a characteristic that forces the dominant areas to always be placed before their dominated areas. Thus, if we divide *n* smallest Z-value areas to *n* partitions, the killer area will be distributed equally to the local Z-Skyline with this optimized shuffling method. Figure 23b presents the effect of optimized shuffling. Each partition has just one killer region and removes dominated areas more efficiently compared to local Z-Skyline using random shuffling. Finally, the global Z-Skyline determines three final profitable areas by examining only four candidate profitable areas.

### 5.5. Complexity Analysis of Distributed Profitable-Area Query Processing

As explained in Section 5.4, distributed profitable-area query processing requires three phases: (1) constructing a profitability map from a selected PQ-index , (2) refining candidate areas by the local Z-skyline algorithm and (3) merging local skyline results to obtain the global answers that are the final profitable areas.

Suppose for a given profitable query, the distributed algorithm is executed by using *N* nodes. We denote the size of the PQ-index as $|PQ_{idx}|$ and the size of local skyline results as $\left|LZSky(PQ_{inx})\right|$.

(11) $$T_{PAQ}=\frac{|PQ_{idx}|\;\times \;\left(T_{PM}+T_{LZSky}\right)}{N}+T_{GZSky}(|LZSky\left(PQ_{idx}\right)\left|\right)$$

Equation (11) describes the complexity of profitable-area query processing, $T_{PAQ}$, where $T_{PM}$ is the average run time to construct the profitability map $T_{LZSky}$ is the average run time to execute the local Z-Skyline, and $T_{GZSky}$ is the average time to perform the global Z-Skyline.

Distributed profitable-area query processing performs better when a killer region exists in the profitability map and the profitable areas in each region are not dense. This is mainly because the killer region enables the algorithm to effectively avoid unnecessary dominance tests; and fewer profitable areas in each region reduces the number of pairwise comparisons when invoking a dominance test.

## 6. Experimental Evaluation

In this section, we present a comprehensive performance evaluation of *DISPAQ* on two real datasets from New York and Chicago, with about 376 million records and 79 million records of taxi trip information, respectively.

### 6.1. Experimental Setup

We implemented our *DISPAQ* system in Java using Java Development Kit (JDK) version 1.7. Spark 1.5 was used as the distributed processing framework and MongoDB 3.2 was chosen for data storage of the PQ-index. All experiments were conducted on commodity machines equipped with an Intel Core i3-6100 3.2 GHz CPU and 8 GB of memory running the 64-bit Ubuntu 16.04 operating system. A total of 5 machines were used as distributed processing clusters for Spark and as data storage nodes for MongoDB. To obtain sound and reliable experimental results, we repeated every test 10 times and averaged all the reported experimental results from all repetitions.

### Dataset

We used a two real taxi trip datasets from New York [62] and Chicago [63] in our experiments. In subsequent discussions, these datasets will be referred to as “NewYork dataset” and “Chicago dataset”. Each dataset contains 30 months of taxi trip data from 1 January 2014 to 31 June 2016.

The NewYork dataset was collected and provided by the New York City Taxi and Limousine Commission. It provides accurate time and GPS coordinates of pickup and drop-off events and consists of 474,000 taxi trips per a day from 13,000 taxis. The total size of NewYork dataset is 56.3 GB.

The Chicago dataset was provided by the Department of Business Affairs & Consumer Protection. It consists of more than 100 million taxi rides, dating back to 2013 with an average 300 MB for each month. Compared with the NewYork dataset, the Chicago dataset has several characteristics to avoid privacy issues. First, the pickup and drop-off times are rounded to the nearest quarter of an hour. Second, the coordinates of each trip are represented as the center coordinates of a census tract and community area. Third, relatively infrequent taxi trips were removed and only frequent taxi trips are maintained in this dataset. Thus, we used the different interval times as explained in Equation (1).

### Queries

Based on the average number of taxi trips in New York City [64], we chose four time categories from a 24-hour day: (1) night time (00:00–04:00), (2) morning rush hour (06:00–10:00), (3) normal hours (12:00–16:00) and (4) evening rush hour (18:00–22:00). For each time category, we randomly generated (area, time) pairs and executed profitable-area query processing.

### 6.2. Experimental Results

In this subsection, we analyze the performance evaluation of *DISPAQ* and compare the profitable-area query processing method of *DISPAQ* with existing approaches. Our goal is to show that *DISPAQ* organizes taxi trip data into a PQ-index very well and efficiently executes profitable-area queries in a distributed way.

#### 6.2.1. PQ-Index Construction

To understand the efficiency of *DISPAQ*, we evaluated the performance of the distributed PQ-index construction. To evaluate indexing performance, we measured (a) the elapsed time of each sub-process and the total wall clock time to build a PQ-index, and (b) the size of the PQ-index by varying the input data size. The length of of the geocode for an area was set to 6, and the length of atime interval was fixed at 10 min for NewYork dataset and at 15 min for Chicago dataset, according to Equation (1).

**Figure 24:**
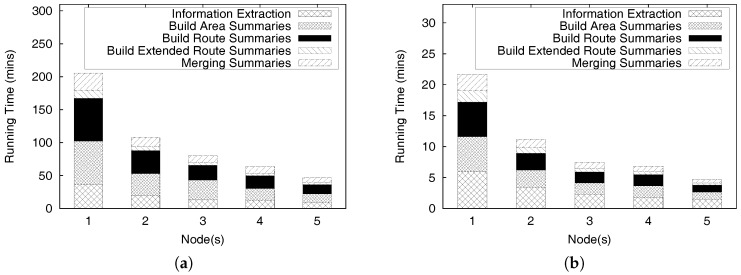
Scalability of the Distributed PQ-index Construction. (**a**) NewYork dataset; (**b**) Chicago dataset.

First, to demonstrate the scalability of *DISPAQ*’s distributed approach, we measured the execution time for constructing a PQ-index by varying the number of commodity servers between 1 and 5. We used 12 months of taxi trips for this experiment. Figure 24 describes the results from constructing the PQ-index in a distributed way. As we expected, the overall time decreases with the number of commodity servers. We observe that processing times from 1 node to 5 nodes decrease linearly. For example, the total execution times for the NewYork dataset changed from 205 min to 47 min, whereas the total execution times for the Chicago dataset decreased from 21.7 min to 4.71 min. In other words, the processing time with *n* commodity servers is almost 1/n of the processing time with a single node. This result corresponds to the complexity cost in Equation (8), which shows that *DISPAQ* inherited the scalability properties of the underlying Spark framework. Another observation is that building area summaries and building route summaries are the most time-consuming processes when a lot of repeated taxi trips exists.

**Figure 25:**
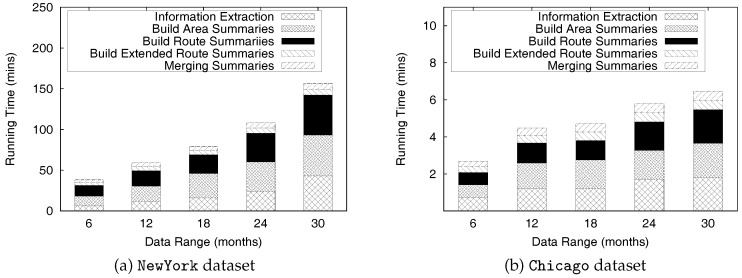
PQ-index construction performance from varying months.

Next, we conducted experiments by varying the data range from 6 months to 30 months in steps of 6 months. The number of machines was fixed to 4. Figure 25a,b summarize the performance to create a PQ-index on both datasets. As expected, the total execution time increases as we increase the size of the dataset by varying the number of months. This is mainly because the number of taxi trips also increases as we vary the dataset size. Another observation is that the construction time of the PQ-index from NewYork dataset takes much longer than for the of PQ-index from Chicago dataset. For example, when *DISPAQ* constructs a PQ-index from 30 months of taxi trips, it takes about 150 min for NewYork dataset and about 6 min for Chicago dataset. The sizes and qualities of both datasets account for the wide difference.

Table 3 and Table 4 show the distributed data flow among sub-processes explained in Algorithm 1. Since the information extraction process removes unnecessary attributes from taxi trips, the size of a shuffle write is almost half of the input data size. *DISPAQ* constructs area summaries and route summaries from the same input data; thus, the sizes of a shuffle read for both sub-processes are the same. Since extended route summaries are combinations of area summaries and route summaries, the sub-process for computing extended route summaries generates a bigger output than the size of the input. The last merging summaries sub-process finally writes the whole PQ-index to the disks of commodity servers.

**Figure 26:**
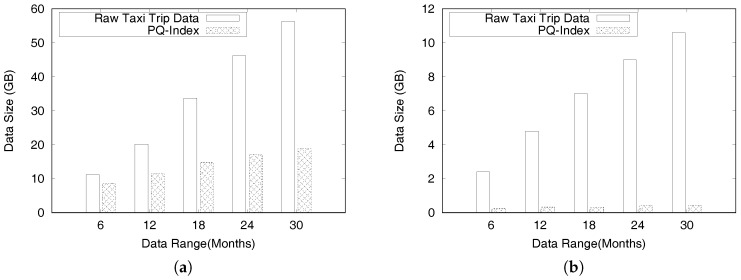
Data size comparison. (**a**) NewYork dataset; (**b**) Chicago dataset.

Figure 26 shows the effective reduction in memory consumption by comparing the size of the PQ-index with that of the raw taxi dataset. The gaps in size widen as we increase the data size by varying months from 6 months to 30 months. This is because we remove unused information and aggregated repeated taxi trips into route summaries and area summaries. Another observation is that the size of the PQ-index from NewYork dataset in Figure 26a is much bigger than that of the PQ-index from Chicago dataset in Figure 26b. The main reason is that Chicago dataset avoids privacy issues by rounding pickup and drop-off times to the nearest quarter of an hour, and by grouping GPS coordinates for pickup and drop-off events.

### 6.3. Distributed Query Processing

In this subsection, we analyze the query performance of the *DISPAQ* system that utilizes a PQ-index.

#### 6.3.1. Query Performance

As explained in Section 5, we implemented distributed profitable-area query processing in two modes: basic Z-skyline and optimized Z-skyline. We also implemented distributed profitable areas query processing based on the traditional skyline approach in two modes: block-nested looping (BNL) and (2) divide-and-conquer (DC). Thus, we randomly chose 10 different profitable-area queries and measured the execution times for these four methods.

**Figure 27:**
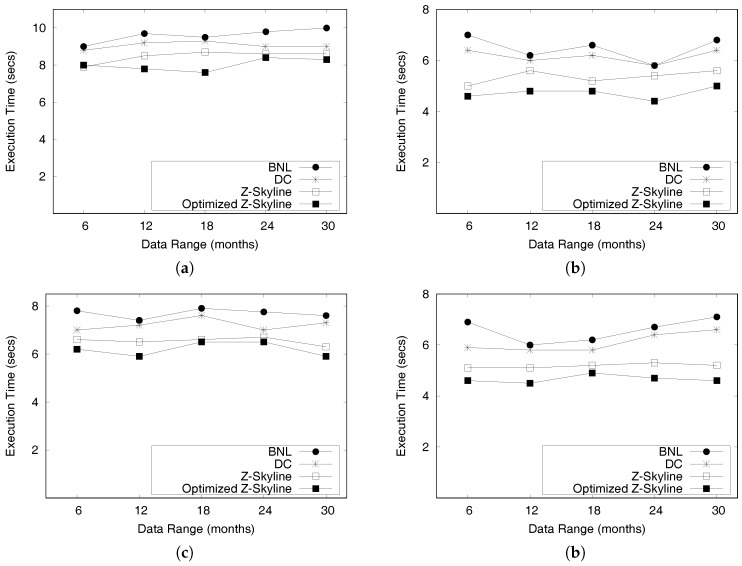
Profitable-area query performance by data range. (**a**) Rush Hour, NewYork; (**b**) Normal, NewYork; (**c**) Rush Hour, Chicago; (**b**) Normal, Chicago.

Figure 27 presents profitable-area query performance. For these experiments, we used 3 machines and varied the data sizes between 6 months and 30 months. Figure 27a,b shows the results for NewYork dataset. We observe that the query execution times for rush hour are slower than normal times. Another observation is that execution times are almost stable, even though the size of the dataset increases according to the number of months. It means the total size of raw taxi trips will not affect query execution time. In all cases, the optimized Z-Skyline method and the basic Z-skyline show better performance than the BNL and DC methods. A similar trend is seen in Figure 27c,d for Chicago dataset.

**Figure 28:**
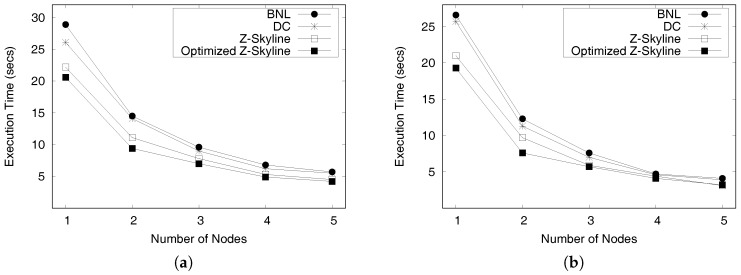
Profitable-area query performance by nodes. (**a**) NewYork; (**b**) Chicago.

Next, we report the evaluation results of the four methods by varying the number of commodity servers. Figure 28 shows the results. As expected, query execution time decreases with the number of commodity servers. Among the four methods, optimized Z-Skyline shows the best performance followed by basic Z-Skyline, and DC. This result indicates the effectiveness of distributed profitable-area query processing based on the Z-Skyline method.

#### 6.3.2. Local Z-Skyline Optimization

In the optimized Z-Skyline, we maximized the number of results in a local Z-Skyline that qualifies in a global Z-Skyline and minimized areas that will be dominated in the global Z-Skyline. We use Equation (12) from Chen et al. [55] to measure the optimality of the local Z-Skyline, where *N* is the number of nodes, $sky_{i}$ is the local Z-Skyline results (candidate profitable areas) in node *i*, and $sky_{global}$ is global Z-Skyline results (final profitable areas).

(12) $$LocalSkylineOptimality=\frac{1}{N}\sum_{1}^{N}\frac{|sky_{i}\cap sky_{global}|}{|sky_{i}|}$$

**Figure 29:**
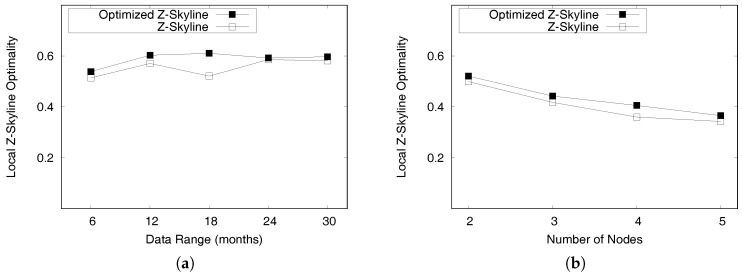
Z-Skyline Optimality. (**a**) Varying the data size; (**b**) Varying the number of nodes.

The values of optimality for both methods are depicted in Figure 29. Figure 29a shows the results when we fixed the number of machines to 3 and varied the sizes of the datasets. The value of the optimized Z-Skyline is always higher than that of the Z-skyline. The optimality values of the optimized Z-Skyline are lowest with 6 months of data; then they increase and become stable after 12 months of data. When we use only 6 months of data, the killing regions in each node are not good enough to dominate other profitable areas, which will be removed during the global Z-skyline. Another observation is that the optimality value of the Z-Skyline is not stable. This is mainly because Z-Skyline uses random distribution when distributing regions to each node.

Figure 29b illustrates the results when we fixed the size of the dataset and varied the number of nodes from 2 to 5. We skipped the optimality value for a single node because it is not meaningful when a single node is used. Since we compute the optimality value by dividing the number of nodes, as defined in Equation (12), the optimality values decreases with the number of nodes. However, the optimality values for the optimized Z-skyline are always higher than the basic Z-skyline.

## 7. Conclusions

In this paper, we address the problem of efficiently retrieving profitable areas when a user poses queries from huge volumes of taxi trip data. We implement a distributed profitable-area query processing system, called *DISPAQ*, by employing Spark and MongoDB. To efficiently obtain candidate profitable areas, *DISPAQ* constructs a hash-based spatio-temporal index, a PQ-index, for maintaining information on profitable areas from raw taxi trip data. *DISPAQ* utilizes a Z-skyline algorithm which considers multiple attributes to refine candidate profitable areas. The PQ-index and the Z-Skyline algorithm enable *DISPAQ* to limit search spaces and avoid a pairwise dominance test among profitable areas during profitable-area query processing. We also suggest an optimization scheme for the Z-skyline algorithm, which efficiently prunes multiple blocks during query processing by distributing killer regions to each node. Performance evaluations on two real datasets demonstrate that the *DISPAQ* approach provides a scalable and efficient distributed solution for indexing and querying huge volumes of taxi trip data. Our experimental results confirm the scalability and effectiveness of *DISPAQ* for processing profitable-area queries.

## Figures and Tables

**Table 1:** Frequently used notations.

Notation	Definition
*T*	a set of taxi trips
ar	area which has a group of locations
rt	route containing a pair (origin area $ar_{o}$, destination area $ar_{d}$)
tp	time period denoted as [start time, end time]
$PA_{ar,tp}$	a profitable area computed from the input area ar and time period tp
$PM_{ar,tp}$	a set of profitable areas computed from the input area ar and time period tp
PA[i]	*i*th element contained in $PM_{ar,tp}$, in other words, a profitable area
$SL(PM_{ar,tp})$	the skyline of $PM_{ar,tp}$ which contains only dominant profitable areas,
	in other words, this is the answer for a profitable-area query
*p*	profit
pd	passenger demand
$t_{cr}$	cruising time
$d_{cr}$	cruising distance
$AS_{ar,tp}$	area summary computed from the input area ar and time period tp
$\mu_{f}$	the average fare
*L*	a list of pickup probabilities
$RS_{rt,tp}$	route summary computed from the input route rt and time period tp
$\mu_{d}$	average distance
$\mu_{tt}$	average travel time
$\mu_{c}$	average expense
$ERS_{rt,tp}$	extended route summary computed from the input route rt and time period tp
$R_{i}$	region which has a set of profitable areas used in the skyline processing

**Table 2:** Taxi Trip Data.

No.	Pickup	Drop-Off	Pickup Location		Drop-Off Location		Trip	Fare	Tip	Tolls
	Date/Time	Date/Time	Longitude	Latitude	Longitude	Latitude	Distance	Amount	Amount	Amount
1	10/16/2015	10/16/2015	−73.98278	40.75492	−74.18142	40.68773	16.63	67	0	0
	10:01	10:23								
2	10/09/2015	10/09/2015	−73.98956	40.75796	−74.18147	40.68773	20.02	70.5	0.5	0
	10:02	10:23								
3	10/16/2015	10/16/2015	−73.9902	40.75703	−73.99946	40.745	1.2	7.5	0	0.5
	10:04	10:11								
4	10/16/2015	10/16/2015	−73.98652	40.75424	−73.99525	40.74455	0.8	6	0	0.5
	10:01	10:09								
5	10/10/2015	10/10/2015	−73.96738	40.80349	−73.95052	40.78425	2	9.5	1	0.5
	10:41	10:45								
6	10/23/2015	10/23/2015	−73.96693	40.80349	−73.95477	40.78422	2.2	9.5	0.5	0.5
	10:42	10:47								
7	10/16/2015	10/16/2015	−73.96551	40.80593	−73.95576	40.78287	2.31	10	0.5	0.5
	10:41	10:46								
8	10/16/2015	10/16/2015	−73.96752	40.80129	−73.96394	40.80769	0.51	4	1	0.5
	10:22	10:26								
9	10/16/2015	10/16/2015	−73.96781	40.80042	−73.96479	40.80662	0.5	4	0	0.5
	10:25	10:30								
10	10/16/2015	10/16/2015	−73.96803	40.80112	−73.95999	40.80827	0.9	5.5	0.5	0.5
	10:21	10:26								

**Table 3:** Distributed data flow of NewYork dataset’s PQ-index construction.

Months	Information Extraction (GB)		Build Area Summaries (GB)		Build Route Summaries (GB)		Build Extended Route Summaries (GB)		Merging Summaries (GB)	
	Input	SW	SR	SW	SR	SW	SR	SW	SR	Output
6	11.2	5.3	5.3	0.04	5.3	2	2	3.9	3.9	8.6
12	20.2	9.4	9.4	0.8	9.4	2.6	2.7	5.4	5.4	11.4
18	33.7	14.7	14.7	0.05	14.7	3.4	3.5	7.8	7.8	14.7
24	46.2	19.7	19.7	0.06	19.7	4.0	4.1	9.3	9.3	17.1
30	56.3	24.1	24.1	0.06	24.1	4.5	4.6	10.4	10.4	18.8

**Table 4:** Distributed data flow of Chicago dataset’s PQ-index construction.

Months	Information Extraction (GB)		Build Area Summaries (GB)		Build Route Summaries (GB)		Build Extended Route Summaries (GB)		Merging Summaries (GB)	
	Input	SW	SR	SW	SR	SW	SR	SW	SR	Output
6	2.4	0.24	0.24	0.0025	0.24	0.14	0.14	0.15	0.15	0.23
12	4.8	0.47	0.47	0.0022	0.47	0.19	0.19	0.22	0.22	0.31
18	7	0.69	0.69	0.0023	0.69	0.27	0.27	0.30	0.30	0.37
24	9	0.88	0.88	0.0024	0.88	0.33	0.34	0.39	0.39	0.39
30	10.6	1	1	0.0025	1	0.35	0.35	0.40	0.40	0.40
